# Tuning ProteinMPNN to reduce protein visibility via MHC Class I through direct preference optimization

**DOI:** 10.1093/protein/gzaf003

**Published:** 2025-03-18

**Authors:** Hans-Christof Gasser, Diego A Oyarzún, Javier Antonio Alfaro, Ajitha Rajan

**Affiliations:** School of Informatics, University of Edinburgh, Edinburgh, EH8 9AB, United Kingdom; School of Informatics, University of Edinburgh, Edinburgh, EH8 9AB, United Kingdom; School of Biological Sciences, University of Edinburgh, Edinburgh, EH9 3JH, United Kingdom; School of Informatics, University of Edinburgh, Edinburgh, EH8 9AB, United Kingdom; International Centre for Cancer Vaccine Science, University of Gdańsk, Gdańsk, 80-822, Poland; Department of Biochemistry and Microbiology, University of Victoria, Victoria, V8W 2Y2, Canada; The Canadian Association for Responsible AI in Medicine, Victoria, V8N 4W7, Canada; School of Informatics, University of Edinburgh, Edinburgh, EH8 9AB, United Kingdom

**Keywords:** protein deimmunization, MHC Class I, protein design, ProteinMPNN, direct preference optimization

## Abstract

ProteinMPNN is widely used in protein design workflows due to its ability to identify amino acid sequences that fold into specific 3D protein structures. In our work, we adjust ProteinMPNN to design proteins for a given 3D protein structure with reduced immune-visibility to cytotoxic T lymphocytes that recognize proteins via the MHC-I pathway. To achieve this, we developed a novel framework that integrates direct preference optimization (DPO)—a tuning method originally designed for large language models—with MHC-I peptide presentation predictions. This approach fosters the generation of designs with fewer MHC-I epitopes while preserving the protein’s original structure. Our results demonstrate that DPO effectively reduces MHC-I visibility without compromising the structural integrity of the proteins.

## Graphical Abstract

**Graphical Abstract:**
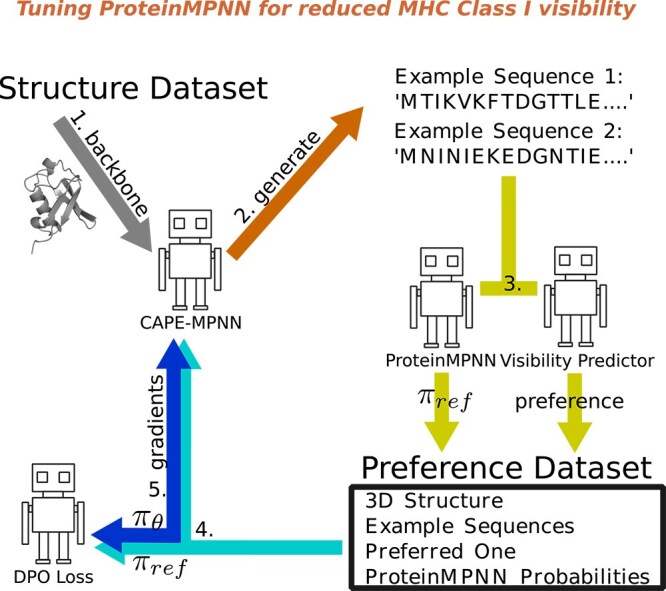

## Introduction

The popular protein design tool ProteinMPNN (Dauparas et al., 2022) tackles the task of predicting amino acid (AA) sequences given a desired 3D protein backbone structure called a template. Some of the proteins designed using ProteinMPNN might be intended as therapeutic proteins [either delivered directly or via messenger RNA (mRNA) or gene therapy], in which case the design process will have to take into account the reaction of the immune-system to these nonself proteins. The consequences of disregarding the immune-reaction could range from reduced therapeutic efficacy up to unwanted auto-immune-reactions targeting the therapeutic protein—as demonstrated by anti-transgene immunity (Carpentier et al., 2015, Mendell et al., 2010).

Deimmunization has a long history (Choi et al., 2013, 2017, Yachnin et al., 2021, Zinsli et al., 2021) and some work has looked into integrating it into modern machine learning (ML) based protein workflows (Bootwala et al., 2022, Gasser et al., 2024, Lyu et al., 2024). However, to our knowledge, our work is the first to directly incorporate this task into ML models like ProteinMPNN, which generate protein sequence based on structure.

The immune-system components that need to be taken into consideration during therapy development will primarily depend on the way the therapeutic is administered and the location where the therapeutic is supposed to unfold its effect. For extracellular proteins antibody (Ab) recognition and presentation by the MHC Class II (MHC-II) pathway will be most relevant. On the other hand, some therapeutics will have to be expressed within human cells, e.g. mRNA therapeutics aimed at mitigating an issue within the cell. In these cases avoiding detection by cytotoxic T-lymphocytes (CTLs) via the MHC Class I (MHC-I) pathway is paramount. To facilitate CTL surveillance, the MHC-I pathway has evolved to present small sub-sequences (peptides, mostly about 8 to 10 AA long) of all proteins expressed within a cell on their surface, which are then detected by the CTLs’ T-cell receptor. These peptides are transported to the cell surface in complex with MHC-I proteins. Each person has up to six different major variants of these proteins, and they present peptides with different motifs. There is high diversity within the population as to the expressed MHC-I protein variants.

ProteinMPNN’s basic functionality is to map the template of a protein’s backbone to AA sequences that are expected to fold into this given geometry. To integrate the goal of reducing visibility (see Definition 1) to CTLs into the design process we tuned ProteinMPNN using direct preference optimization (DPO) introduced in Rafailov et al. (2023). DPO adjusts the base network’s weights to increase the probability of generating sequences that conform better to our preferences. Our contribution is to adapt this tuning technique to ProteinMPNN, generate sequences with reduced immune-visibility, and analyze the trade-off between visibility reduction and sequence quality. We use structural bioinformatics methods to assess the quality of the generated proteins. The result of our work is to release a new method to tune model weights for ProteinMPNN tailored toward MHC-I de-immunization for a particular patient, called CAPE-MPNN.

Definition 1 *MHC-I immune-visibility, immune-visibility, or visibility:* By absolute visibility, we mean the number of 8, 9, and 10-mers within a protein sequence to be presented on the cell’s surface (overlaps are counted separately) to CTLs by the MHC-I pathway. If a k-mer is presented by several MHC-I alleles this increases the absolute visibility number only by one. Because of technical reasons, this contrasts to the definition used in Gasser et al. (2024), where it was increased by the number of MHC-I alleles that presented the k-mer. As different people will regularly express varying MHC-I proteins, it is important to specify the alleles used during the analysis. Throughout the paper we consider a hypothetical patient with HLA-A*02:01, HLA-A*24:02, HLA-B*07:02, HLA-B*39:01, HLA-C*07:01, and HLA-C*16:01 MHC-I alleles.

We use existing computational methods to identify the presented kmers in a sequence. During evaluation we directly rely on *netMHCpan 4.1* (Reynisson et al.  2020) predictions. We accelerate training by approximating MHC-I binding prediction using a standard position weight matrices (PWMs) based method during fine-tuning (see *Predicting presented peptides*).

An example will make the concept of absolute visibility clearer. The 11 AA long sequence GANIWGANNNV holds two 10-mers, three 9-mers, and four 8-mers. A peptide prediction predictor is used to assess each of these nine k-mers for presentation (see *Predicting presented peptides*). In case we were using *netMHCpan 4.1* (with a rank threshold of 2%) and our hypothetical patient, only the kmers ANIWGANNNV and NIWGANNNV would be presented by any of the six alleles. So, the sequence’s absolute visibility is two.

This paper often compares the visibility of a protein sequence with that of a template structure from the Protein Data Bank (PDB). Therefore, we use *relative visibility*, defined as the sequence’s visibility divided by the template’s visibility.

There is an important distinction between a peptide’s immune-visibility and immunogenicity. Being visible on the cell’s surface is a necessary but not a sufficient condition for a CTLs reaction (it does not imply immunogenicity).

### ProteinMPNN workflows

Since they share the same architecture as ProteinMPNN, our CAPE-MPNN model weights can easily replace the original ProteinMPNN weights in existing protein design workflows. Such a workflow might start by the generation of a backbone template with a diffusion model like RFdiffusion (Watson et al., 2023) or FoldingDiff (Wu et al., 2023).

Afterwards, the 3D coordinates generated by these techniques can be used as input to ProteinMPNN to generate AA sequence candidates. An alternative workflow might start with a known protein sequence for which we want to generate other sequences with similar function but different sequence. Deimmunization, where the goal is a protein that is less immune-reactive but functions similarly, is an example of this. In these cases at first the structure of the protein is determined (experimentally or predicted) and then used as input into ProteinMPNN.

Typically, the previous step will propose a set of candidate protein designs. These are usually filtered by a series of bioinformatics methods, ranking them according to the likelihood that they will fold, function, and conform to our preferences. This assessment might be computationally very costly, e.g. if molecular dynamics simulations are involved, which can typically take days to run. It is, therefore, preferable that the generated sequences are already enriched in a desirable property, and stand a good chance to pass these downstream filters.

### Tuning ProteinMPNN for less visibility

As is in its name, ProteinMPNN is a message passing neural network (MPNN). It can be subdivided into a *Backbone Encoder* and *Sequence Decoder*. The encoder updates the initial node embeddings (each AA is modeled as a node) and edge embeddings (model the relationship between a node and one of its nearest neighbors). The decoder then predicts the AA sequence in an auto-regressive (AR) fashion. This means that, given the sequence created up to this point, the model produces a probability distribution over all possible AAs for the next AA in the sequence. For this the decoder can access the edge, node, and token (of residues predicted so far) embeddings. The training objective was to minimize categorical cross-entropy per predicted AA sequence token. While the standard procedure to generate text with AR models would be to use the standard left to right order within text, ProteinMPNN was trained to generate sequences in an arbitrary order (e.g. start with position 10, then sample position 50, 5, 23,...). A detailed description of the information flow within the model can be found in *Appendix: ProteinMPNN information flow*.

The way the *Sequence Decoder* generates tokens is very similar to language models (LMs). This raises the question of whether similar fine-tuning techniques as for LMs can also be successfully applied to ProteinMPNN. Analogous to chatbots, where we want to discourage the usage of certain words and phrases by the model, we want the model to avoid the usage of presented peptides to reduce immune-visibility. Therefore this paper explores using a method developed for LM alignment—namely DPO—to the task of protein deimmunization with ProteinMPNN.

### Direct preference optimization

This technique is typically applied to large language models (LLMs) to improve their harmlessness and usefulness. It is derived from the more general method of reinforcement learning from human feedback (RLHF) (Ziegler et al., 2020), which was also used to tune the GPT foundation models to produce the first versions of ChatGPT.

At first a foundation model (like OpenAI’s GPT-3) would be trained using self-supervised learning (e.g. on a corpus of text from the internet). Next word prediction is a common training objective. This foundation model will then be able to generate likely text continuations, which are, however, not focused on a particular task—like answering questions, helping write text, summarizing text. Typically, function-specific data are then used to fine-tune the model to these tasks. However, after this step, there might still be a lot of dangerous language remaining. To tune the model further, RLHF was introduced.

RLHF is based on examples generated by the fine-tuned foundation model. These examples are then rated by humans. A typical way to do so is to let a person select a preferred response to a prompt out of two options. Alternatively, a person could also assign ratings to each response. Independent of the concrete procedure, a reward model can then be trained in a supervised way based on these feedbacks. This reward model predicts higher rewards for responses that are predicted to be preferred by humans. RLHF then relies on a reinforcement learning training step—typically using Proximal Policy Optimization (Schulman et al., 2017)—for which the reward model provides the rewards.

Tuning a model using RLHF can be quite difficult in practice. Therefore, a more straightforward way to incorporate preferences into LLMs had to be found. For the RLHF version in which the feedback consists of humans choosing their preferred response from two generated ones, a more direct way is introduced in Rafailov et al. (2023). The authors show that this particular RLHF setting optimizes for the same objective as a supervised fine-tuning task they called DPO.


(1) \begin{align*}& \mathcal{L} = - \mathbb{E}\left[\log \sigma\left(\beta \log \frac{\pi_\theta(y_{w} | x)}{\pi_{ref}(y_{w} | x)} - \beta \log \frac{\pi_\theta(y_{l} | x)}{\pi_{ref}(y_{l} | x)}\right)\right]\end{align*}

The DPO training objective is to minimize the loss in Equation 1 (Rafailov et al., 2023). $x$ is the prompt for the generation (in our case the backbone template we want to generate a sequence for). $y_{w}$ is the preferred response (generated AA sequence), while $y_{l}$ is the less preferred one. $\pi _{ref}$ denotes the density function of the original model and $\pi _\theta $ that of the tuned model. An important parameter is $\beta $, which controls how far we want the model to steer from the original one.

For the purpose of training CAPE-MPNN, ProteinMPNN plays the role of the foundation model, while the role of human feedback is played by an MHC-I presentation predictor (see *Alignment*).

## Methods

This section first explains how we predict presented peptides within a sequence. Then the used data sources are introduced. Afterwards the details of aligning the model to our immune-visibility target are laid out. This is followed by how new designs were generated. We then discuss the metrics utilized to assess the quality of the designs and compare the various model checkpoints. Eventually, the trade-off analysis is outlined, which resulted in the identification of promising DPO hyper-parameter sets.

### Predicting presented peptides

Our concept of visibility (see Definition 1) involves identifying all the 8–10mers within the protein sequence presented on the cell surface. *netMHCpan 4.1* (Reynisson et al., 2020) is a very commonly used tool for this task. The algorithm takes a peptide and an MHC-I allele as input. *netMHCpan 4.1* outputs a score (indicating likelihood of cell surface presentation) and a rank, comparing the peptide’s score with random natural peptides. A rank of 2% means that the peptide’s score was higher than the top 2% of scores from random natural peptides. In general, a rank threshold of 2% is considered to include also “weak binders”, while a threshold of 0.5% would be considered to only correspond to “strong binders” (Reynisson et al., 2020).

Unfortunately, obtaining *netMHCpan* predictions on the fly during training is very slow. So, **for alignment purposes only (not during evaluation)**, we constructed fast PWM-based classifiers. Each PWM has one row per AA and one column per position in the peptide. The usage of PWMs is standard practice in bioinformatics. In our case, the matrix values represent the log-likelihood probabilities of locating the AA, corresponding to a specific row, at the position, corresponding to a specific column in peptides presented by the MHC-I pathway. To find these probabilities, we first sampled three million random 8–10mers. Then, we used *netMHCpan 4.1* with a rank threshold of 2% to assess whether these were presented by any of the six MHC-I alleles of the patient (all six alleles are represented by a single PWM). The probability of finding, e.g. a leucine at position four of a 9-mer would then be the percentage of predicted presented 9-mers that have a leucine at position four (e.g. 4.2%). Presentation of a new peptide is then assessed based on the sum of these log-likelihood probabilities being higher than a threshold. This threshold is determined by first finding the percentage of random peptides of a certain length that are predicted by *netMHCpan* to be presented by the patient (e.g. 19.6%)—the threshold is then the number that makes the PWM classifier predict as many presented peptides in the random set as *netMHCpan*. In *Appendix: PWM presentation predictor example* we walk through a concrete example.

To reiterate, **for evaluation purposes**, we directly use *netMHCpan 4.1* to identify the sequence’s presented peptides.

### Data

We use three datasets. The first one (*General proteins*) is obtained from the ProteinMPNN dataset and we use it for fine-tuning and validation. The second one (*Specific proteins*) is a selection of specific proteins we randomly selected from the original ProteinMPNN dataset to facilitate more compute intense performance comparisons—in particular based on the predicted structure of designed sequences. The final one is a set of *Illustrative proteins*, which might be particularly interesting for the community.

#### General proteins

We use the same data as were used in the original ProteinMPNN paper (https://github.com/dauparas/ProteinMPNN/tree/main/training), keeping their training, validation, and test split. The generation of examples from this dataset can be best thought of in two steps—the *PDB Dataset* and the *Structure Dataset*.

#### PDB dataset

The examples are based on PDB crystal structures with a resolution cutoff of 3.5 Å. The initial dataset consists of 473 062 AA chains (106 344 unique AA sequences). PDB chains were clustered at 30% sequence identity using mmseqs2 (Steinegger and Söding, 2017)—so each cluster consists of similar chain-sequences. This should ensure that no sequence information is leaked from the training to the validation set. These were split into 23 349 training, 1464 validation, and 1539 test clusters. An epoch iterates over all clusters, producing one example per cluster. When an example is requested, first a chain is sampled from the cluster using a uniform distribution. This chain is linked to a single PDB entry and the biological assemblies that the chain is part of within this entry are identified. If it is not part of any, then the chain alone is used. If it is part of several, a random biological assembly is picked. Then all the chains in the selected biological assembly are loaded and their coordinates transformed via the biological assembly specific transformations (chains can be in different positions and/or orientations in different biological assemblies). Many proteins consist of similar chains (e.g. homo-dimers). To prevent leakage of sequence information from one chain to another during training, masking (no sequence information is provided) of all chains with a pairwise TM-score above 0.7 with respect to the selected chain is enforced—these then have to be designed by the model. For the purpose of generating the preference data, we always require the model to design all chains in an assembly.

#### Structure dataset

The PDB Dataset described above is then used every two epochs to generate a refreshed Structure Dataset with different chains sampled from the clusters. This will have less examples, since in this step the maximum sequence length of the biological assemblies is restricted to 10 000 AAs (next to other drop-outs, $<10\%$ shrinkage total). In this step they also remove leading or terminating histidine repeats. The batches supplied to the model are yielded from this dataset. A single batch has a maximum of 10 000 residues (see Table 1). This can be filled with a single very long biological assembly, or several smaller ones. This arrangement makes graphics card memory overruns less likely. Each of these sequences can comprise of multiple chains. We will refer to the number of sequences in a batch as $B$ and the maximum length of any of those sequences as $S$. It holds $B \cdot S_{max} < T_{max}$.

**Table 1:** ProteinMPNN hyper-parameters.

Hyper-parameter	Symbol	Value
Vocabulary size	V	21 (20 AA + “X”)
Feature dimension	D	128
Nearest neighbors	N	48
Max number of tokens per batch	$T_{max}$	10 000
Maximum sequence length	$S_{max}$	10 000

The way this information is handed over to ProteinMPNN is laid out in detail in *Appendix: ProteinMPNN data embeddings*.

#### Specific proteins

To identify proteins for more compute intense analysis (based on predicted structure), we selected some specific proteins from the original ProteinMPNN dataset. The six specific validation proteins (IDs: 1B9K, 1OA4, 1QWK, 1TJE, 1XGD, 4RQG) were randomly sampled [only X-Ray crystallography PDB IDs of monomers (consisting of only a single AA chain) and homo-oligomers (consisting of multiple AA chains with the same sequence), chains need to be longer than 50 AAs, entries cannot include >2000 AAs, we also exclude entries with small molecules or DNA strings as well as entrances with more than five unmodelled AAs] from the original validation set and used in the trade-off analysis (see *Trade-off analysis*). Similarly, the 10 specific test proteins (IDs: 1A3H, 1P3C, 1PGS, 1QKD, 1S5T, 1X0M, 2BK8, 3O6A, 3TIP, 3WOY) were randomly sampled from the original test set. Here, to provide the reader with an expected range for the values of the assessment criteria, we gave priority to the PDB IDs for which we could identify peer groups. These peer groups were identified by performing a “strict_shape_match” on the PDB and removing all results with a score above 99% (to remove identical entries). We also restrict the peers to having a TM-score of above 90% with the template structure. The peer and template sequences must not be exactly the same and their lengths cannot differ by >10%. The search was stopped after finding 10 peers and if there are less than three, the entire peer group is disregarded.

#### Illustrative proteins

In addition, we chose five well-known proteins to show how our method performs on these. The proteins selected were TEM-1 (1M40), ubiquitin (1UBQ), ferritin (1SQ3), GB1 (2QMT), and green fluorescence protein (4KW4). These manually added proteins are highlighted in bold and are plotted on the left side in all multi-protein figures. We do not ensure that these are unseen during training or validation.

### Alignment

The alignment process is meant to adjust a base model’s weights in a way that causes its responses to better conform to our preferences. In a natural language processing (NLP) setting the prompt might be a question to which the LLM is supposed to respond with a helpful and harmless answer. In contrast, in our protein design setting the prompt is a protein backbone template, to which the model responds with a low immune-visible AA sequence. The basic hypotheses of this work is that the DPO process as used in a NLP setting can also be used in the protein design setting to generate designs with low visibility while retaining the same structure.

As a base model for the alignment, we use the v_48_020 version of ProteinMPNN with the hyper-parameters in Table 1.

As seen in *Direct preference optimization*, DPO tuning requires the creation of a preference pair dataset. This is made up of examples consisting of a prompt (in our case a 3D backbone structure), two possible responses generated by the model as well as a flag signifying which of those is preferred. To generate this, we adopt the following procedure.

- For a backbone example in the general training data (see *General proteins*), we let the current aligned state of CAPE-MPNN generate two candidate designs. We require the network to generate all AAs within each sequence.
- Although DPO is based on RLHF, which has “human feedback” in its name, we replace this by feedback from bioinformatics methods that assess the immune-visibility of generated sequences. Less visible ones are preferred. In this first iteration of CAPE-MPNN we only consider visibility via the MHC-I pathway. Although we use the same concept of visibility during training of CAPE-MPNN as well as evaluation of the designed proteins (see Definition 1), we use different prediction tools to find the presented peptides within a sequence. During evaluation we directly apply *netMHCpan 4.1*. This, however, can be extremely time-consuming during training. Here, we resort to the faster PWM-based method described in *Predicting presented peptides* for finding the preference dataset example with the preferred visibility.
- In addition, we also use the original ProteinMPNN weights to calculate the likelihood of the two examples being generated as responses to the backbone as this is required constant information in the DPO loss function (see Equation 1).

With this preference dataset, the model is then aligned for two epochs. Then a new preference dataset is generated using the latest aligned version of CAPE-MPNN. In contrast to the original DPO paper (Rafailov et al., 2023), which uses the base model to generate the preference examples, we use the tuned one. The motivation for this is that while in language tasks an answer is typically either acceptable or not, in our task we want to move the model further and further away from the original outputs to gradually reduce immune-visibility. Therefore, we think that it is necessary to generate preference examples that reflect the current state of tuning. However, the $\pi _{ref}$ used for the loss (Equation 1) remains the one of the ProteinMPNN base model.

### Design generation

When designing *General proteins*, we sample each design only once. In contrast, for *Specific proteins* and *Illustrative*  *proteins*, we generate three candidate protein designs (using a temperature of 0.1) from which we select the best one (see below). Since we observed that some of the checkpoints would output only constant chains, we remove all candidate sequences that include <10 different AA types. We then use ColabFold (Evans et al., 2021, Jumper et al., 2021, Mirdita et al., 2022) to generate 3D structures for each of the remaining candidate designs (unsuccessful designs get assigned a TM-score of zero). In case there are candidates with TM-scores above 0.9, we select the one with the minimum visibility from those. Otherwise, we select the candidate that has the maximum TM-score as the finally designed sequence.

### Assessment criteria

The assessment of the protein sequences is based on third-party bioinformatics tools. In some cases we directly report their values (e.g. TM-score), while in other cases we process them further (e.g. ‘visibility’). This subsection lists the tools used and how their outputs were processed further. For the template proteins of PDB, we already have a pdb file. To obtain 3D structures for designed sequences, we used ColabFold, which is based on AlphaFold 2 (Evans et al., 2021, Jumper et al., 2021, Mirdita et al., 2022).

- **Visibility:** during evaluation, the visibility assessment of a sequence is based on Definition 1 in combination with the *netMHCpan 4.1* predictor and a 2% rank cutoff. (see *Predicting presented peptides*). A 2% rank cutoff is very conservative. In general, only 1 in 200 peptides can be expected to bind to a given MHC-I allele (Yewdell and Bennink, 1999). This would correspond to a 0.5% rank cutoff. We therefore expect the absolute visibility of the sequences to be lower. However, we will normally not use the absolute visibility from Definition 1, but rather compare the visibility of the design with that of the sequence from the protein template in the dataset. So if the designed protein has a visibility of 9, while the template had 36, the design’s visibility will be scored as 0.25.
- **Sequence recovery:** similar to the original paper (Dauparas et al., 2022), we also look at the percentage of AAs that are the same in the natural protein used as template as in the generated sequence.
- **TM-score:** a common method to compare the structural similarity between two proteins is the TM-score. This can be calculated with *TMalign* (Zhang and Skolnick, 2005). TM-scores take values between zero and one. While scores below 0.2 are associated with random, unrelated proteins, scores that are higher than 0.5 point to the same fold in SCOP/CATH (https:/seq2fun.dcmb.med.umich.edu/TM-align/).

In addition to these metrics for immune-visibility reduction and similarity, we used *DESTRESS* (Stam and Wood, 2021) to obtain the following additional information about the original template proteins as well as our designs.

- **DSSP edit distance:** alongside *TM-score*, this indicator offers an alternative perspective on structural similarity—based on the notion that protein secondary structure should be conserved. Given a pdb file, the DSSP program (Kabsch and Sander, 1983, Touw et al., 2015) assigns one of eight secondary structure elements to each AA in this file. Each element is represented with a character (see https://swift.cmbi.umcn.nl/gv/dssp/DSSP_2.html). For each design we compare the resulting string with the string produced for the template. The *DSSP edit distance* is then the length standardized edit distance between those strings.
- **Rosetta total:** thermodynamics dictates that proteins will spend more time in lower energy folding configurations than in higher energy configurations. Therefore, in general a lower folding energy is considered favorable. However, Rosetta calculates a biomolecule’s energy by “scoring” it. This energy scoring calculation does not solely rely on physical terms but also statistical ones (see Burman and Mulligan, 2017). Therefore, Rosetta uses Rosetta Energy Units (REUs) instead of standard units for energy and there is no direct conversion from REU to kcal/mol. Rosetta returns the score for the whole structure. However, to compare across different protein lengths, we examine the score per AA. A typical score for a refined structure is in the range of −3 to −1 REU per AA (Alford et al., 2017, Burman and Mulligan, 2017).
- **
$\Delta $ Hydrophobic fitness:** the *Hydrophobic Fitness score* developed in Huang et al. (1995) assesses how nonpolar the environment of hydrophobic residues (they use C, F, I, L, M, V, and W) in the protein is and how well they are buried within the protein. We subtract the template’s score from the design’s score to arrive at *$\Delta $ hydrophobic fitness*. Lower scores and therefore also deltas are considered better.
- **Packing density:** we use the packing density number provided by Stam and Wood (2021), which is based on the ISAMBAD (Wood et al., 2017) function. To calculate this, first the number of (non-hydrogen) atoms within 7 Å of each atom is calculated. A protein’s *packaging density* is then the average of this number over all atoms in the structure. A higher value is considered better.
- **
$\Delta $ aggrescan3d avg/max:** one of the major limitations for the production and storage of soluble therapeutic proteins is aggregation. This can be driven by the exposure of regions with hydrophobic residues on the protein’s surface. For each residue aggrescan3d (Kuriata et al., 2019) calculates an aggregation score. The lower this score the better. We compare its average as well as its maximum value across the sequence to the naturally occurring template protein’s values.
- **Isoelectric point:** the higher the concentration of $H+$ is in a solution (lower pH), the more of these will bind to the protein. This makes its charge more positive. So for very high pH values the protein will be negatively charged. As the pH falls, more and more protons will bind to it and increase its charge. The isoelectric point (pI) is then the pH of the solution at which the protein has neutral charge.

### Trade-off analysis

To analyze the trade-off between visibility reduction and quality of the generated proteins, we conduct a DPO hyper-parameter search. The trade-off analysis is based on 20 alignment runs over 20 epochs each. Each alignment run (see *Alignment*) was performed with a different randomly sampled hyper-parameter set (see Table 2). Of these runs, three failed due to stability issues (possibly due to too high $\beta $ values). Of the succeeding runs, we selected the checkpoints after epoch 2, 10, and 20 for further analysis (17x3 checkpoints to compare).

**Table 2:** **CAPE-MPNN DPO hyper-parameter** distributions for the trade-off analysis and values for two selected fine-tuning configurations.

DPO	Distribution	Value	
hyper-params		458340e4	e32b8ed0
beta	log uniform(0.001, 0.2)	3.43e-2	4.48e-3
temperature	uniform(0.01, 1.0)	0.759	0.500
learning rate	log uniform(1e-7, 1e-3)	2.86e-6	4.62e-7

Since running a visibility analysis for the whole general validation set (see *General proteins*) for all model checkpoints would take too long time, we only randomly selected 98 examples to estimate the expected visibility (mean visibility) and the expected sequence recovery (mean sequence recovery). Calculating TM-scores takes even longer. So we only use the six specific validation proteins (see *Specific proteins*) for estimating the expected TM-scores (mean TM-score).

### Specific and illustrative protein analysis

**Figure 1:**
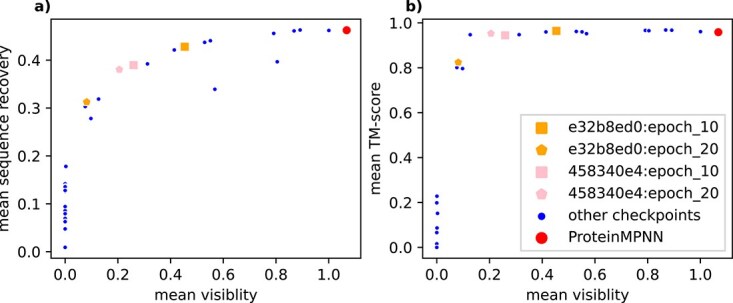
Trade-off between visibility and structural validity on validation data: each data-point in these two plots represents a checkpoint. The red one is the original ProteinMPNN network. The blue ones represent checkpoints that were created during the hyper-parameter search (after 2, 10, and 20 epochs). The orange and pink ones are for two selected hyper-parameter combinations. Both plots show the mean relative visibility to MHC-I over the general validation proteins on the x-axis. (a) shows the mean sequence recovery over the general validation proteins and (b) shows the mean TM-score over the specific validation protein designs on the y-axis.

Finally, based on the kink observed in the mean TM-score in Fig. 1b, we selected two promising models identified in the trade-off analysis (458340e4 and e32b8ed0, see Table 2) to analyze their performance on specific test and illustrative backbones. We analyze the resulting individual designs with regards to the criteria set out in *Assessment criteria*.

## Results and discussion

This section first presents the results of the trade-off analysis that was carried out using validation set backbone structures. Afterwards, we look into the performance of two models on specific backbone structures in more detail.

### Trade-off analysis indicates high-quality designs for up to 70% visibility reduction

To analyze the dependence of quality of generated sequences (as measured by TM-score) and visibility modification achieved, we tuned CAPE-MPNN checkpoints with various hyper-parameters (see Table 2).

Figure 1, which plots the visibility modification vs. the quality of the designs, shows that there exists a trade-off between those two objectives. Both plots in the figure show that the ProteinMPNN base model (v_48_020) designs in red are of high quality but with similar visibility to the natural template proteins. In comparison, the CAPE-MPNN designs experience a trade-off between quality and visibility. The ideal structures would be found in the top left corners of Fig. 1. In contrast, the more the visibility is reduced, the lower the predicted quality.

Figure 1a shows that the reduction in sequence recovery, which is linked with a reduction in visibility, seems to be quite steady and there seems to be a narrow band of attainable recovery values for a given visibility. This points to most checkpoints being pareto optimal (one cannot improve on one metric without worsening another) with respect to the visibility/quality trade-off—independent of the actual hyper-parameters used for fine-tuning them. In comparison, Fig. 1b depicts that the predicted TM-scores stay quite high before suddenly falling off a cliff at a level of roughly 20% the original visibility.

**Figure 2:**
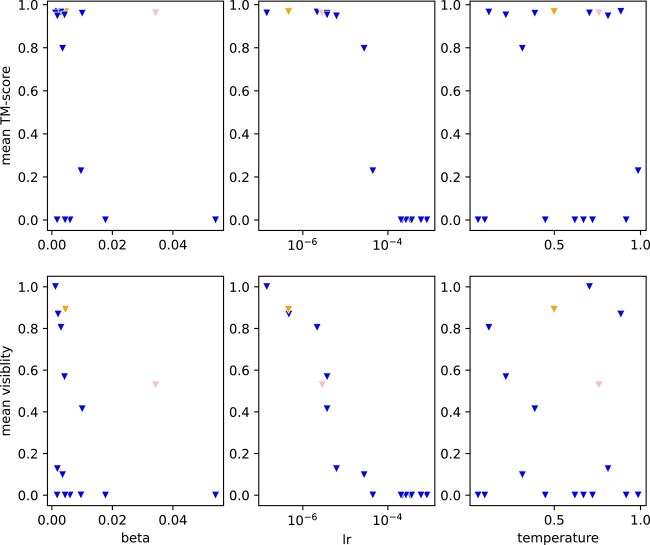
Influence of DPO-hyperparameters on the quality vs. visibility modification trade-off: each triangle in the plots corresponds to a single checkpoint (after epoch 2 of tuning). The colors are the same as in Fig. 1. Each column of the figure shows the influence of a variation in a DPO-hyperparameter. The first row depicts the influence on the mean TM-score of the designs for the specific validation backbones. The second row depicts the influence on the mean visibility of the designs for the general validation backbones.

To identify which hyper-parameters determine the eventual position of a model on this trade-off frontier, Fig. 2 depicts the dependence of TM-score and visibility on the three varying hyper-parameters (Table 2) after two epochs of tuning. We found that high beta values would lead to unstable training (not depicted in the figure). Surprisingly, we do not see a clear relationship between $\beta $ and visibility reductions. Learning rate (lr) seems to have an influence on the eventually achieved quality and visibility modification. Unsurprisingly, high learning rates seem to “destroy” the ProteinMPNN weights and make the model output gibberish. In contrast, very low learning rates do not modify the weights at all. Raising learning rate then first seems to affect visibility in a positive way before leading to reductions in TM-score. The temperature used to sample the preference examples seems to have little influence on the eventual outcome.

### Specific and illustrative protein analysis reveals differences in the quality of proposed sequences

**Figure 3:**
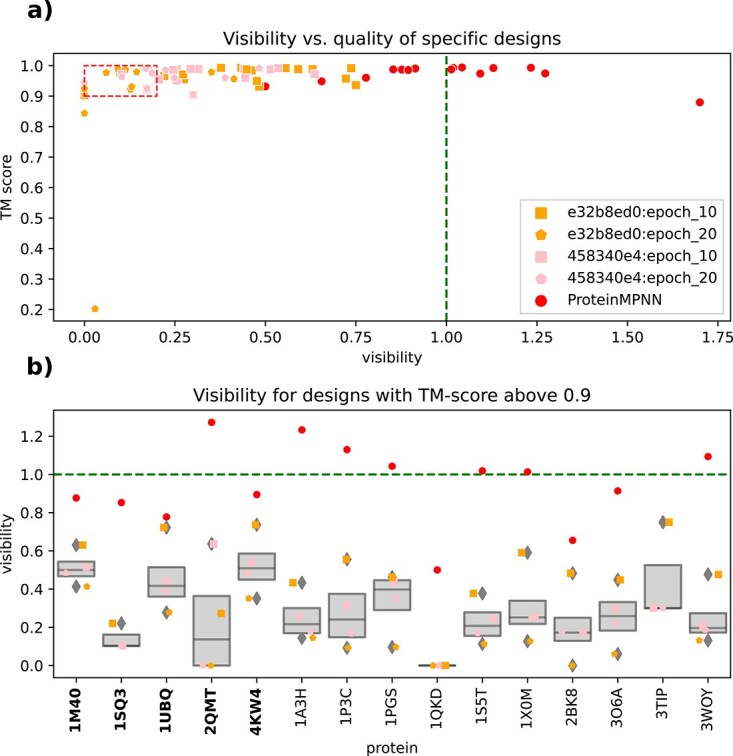
A broad performance range: this figure looks into the predicted structural validity and visibility reductions of specific test and illustrative (in bold on the left side) designs. In plot (a) the quality of designs (as measured by TM-score on the y-axis) gets compared with the relative visibility (x-axis) for two DPO hyper-parameter selections after two epochs of tuning. Each point represents a design for a template using the checkpoint. We find that most designs have high TM-scores above 0.9. Not all of them are less visible than the original sequence (visibility $< 1.0$) and we also see that the ProteinMPNN designs in red tend to have far higher visibility. Furthermore, some designs also only show a maximum of 20% the number of visible peptides in the designs (red dashed box). In plot (b) we then look into the distribution of visibilities of designs. These are a subset of designs from plot (a) that satisfy the condition that their TM-score is above 0.9. The dashed green lines represent the visibility of the PDB proteins. The same visualization for the specific validation set can be found in Appendix Figure A1.

With regards to specific and illustrative proteins (see *Data*), we observe a broad range of performance (Fig. 3). There are some proteins for which the results are quite encouraging. In particular, we find that 458340e4:epoch_20 and e32b8ed0:epoch_20 designed candidates for GB1 of *Staphylococcus aureus* (PDB: 2QMT) that have a predicted visibility of zero, while the predicted TM-score retains a high value of above 90%. In contrast, we see that the ProteinMPNN base model in Fig. 3a regularly generated candidates with over half the template’s visibility and it never generated a zero visibility design.

To gain a more comprehensive insight into the properties of the designs, we used DESTRESS (Stam and Wood, 2021) that provides a variety of indicators (see *Assessment criteria*). To get a feeling for the range of values to expect for these, we identified peer proteins for the natural template proteins (see *Specific proteins*). The range of values taken by these peer groups can be found as green bars in the background of Fig. 4, which presents the various indicators obtained using DESTRESS (see *Assessment criteria*). The same information is also displayed for each individual specific test and illustrative protein in Fig. 5 and the corresponding **Figures A3, A5, A7, A9, A11, A13, A15, A17, A19, A21, A23, A25, A27, A29, A31, A33, A35, A37, A39, A41** in the *Appendix: Protein assessment details*.

**Figure 4:**
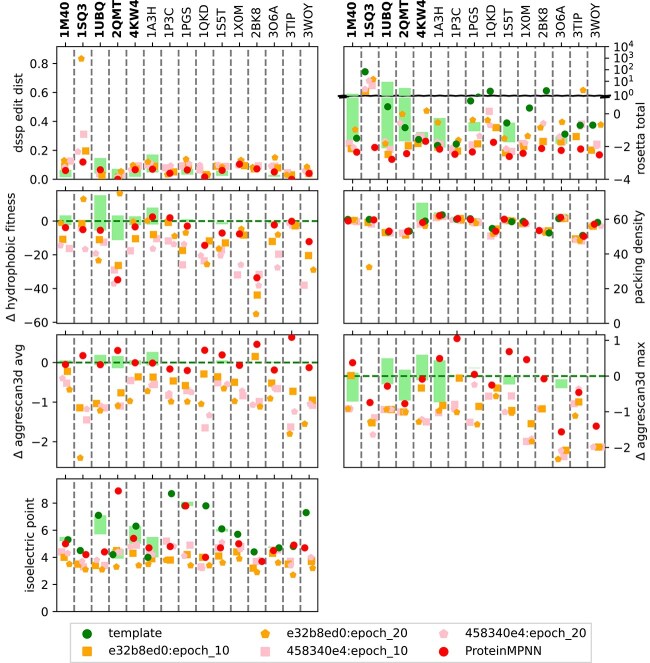
Quality indicators for designs: the figures above show the distribution of quality indicators for the ProteinMPNN and CAPE-MPNN designs by specific test and illustrative (in bold on the left side) protein templates. Each dot signifies a design. For 1M40, 1UBQ, 2QMT, 4KW4, 1A3H, 1PGS, and 1S5T the green bars in the background depict the range of values for identified naturally occurring peer proteins (see *Specific proteins*). For all indicators except packing density and pI, lower values are considered favorable. With regards to pI, we want to be as close as possible to the template protein and with regards to packing density, higher values are considered favorable (Stam and Wood, 2021). The figure showing ”rosetta total” is split in two parts. The bottom part (range −4 to 1) depicts the normal range of values. To also depict the outliers that have problematic values we also show a second log scale above. The same visualization for the specific validation set can be found in the Appendix Figure A2

**Figure 5:**
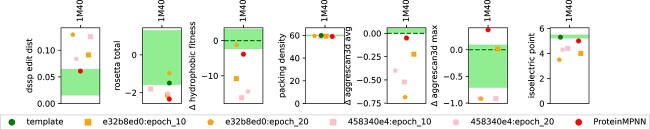
Stable designs with reduced isoelectric point: this figure depicts the same information as Fig. 4, but focusing only on 1M40.

**Figure 6:**
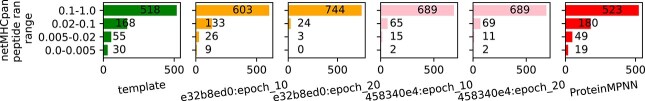
Increased netMHCpan peptide ranks for tuned networks: the bars show the number of 8–10-mers within the chain that have a *netMHCpan* rank within the stated range. The lowest category (0.0–0.005) corresponds to ”strong binders”, the second lowest category (0.005–0.02) to ”weak binders”, while the other categories are predicted not to be presented. We find that the tuned models have far fewer kmers in the two lowest categories—pointing to a reduced immune-visibility.

**Box 1:**
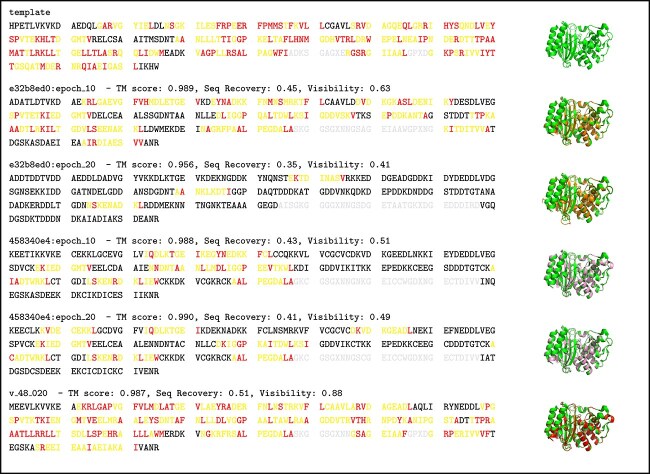
Template and designed sequences for 1M40: the sequences depicted here belong to the points shown in Fig. 5. AAs printed in black are not present in any predicted epitope (*netMHCpan* ranks of all 8-10mers this AA belongs to are above 2%). AAs printed in $yellow$ are part of at least one predicted presented peptide. AAs printed in $red$ are anchor AAs of at least one predicted presented peptide (second or terminal AA of the peptide). Finally, the presentation of AAs in $gray$ is unclear, since they are in the vicinity of unknown AAs (X). Next to the sequences, there are figures showing the predicted 3D protein structure aligned to the template protein (1M40 in this case). The template is colored in green and the designed protein has the same color as in Fig. 5. In case there are several copies of a chain within the biological assembly, we only show the first of those.

The foreground of Fig. 4 displays colored dots, where each dot represents a single design produced by a checkpoint. In general, the designs show promising results, although some individual ones (in particular from e32b8ed0:epoch_20) can have unfavorable values for some protein designs.

- For the *DSSP edit distance*, we find that most designs fall comfortably in the range of their respective peer group. However, e32b8ed0:epoch_20 sometimes produces designs that seem far away in structure space, indicating failed designs.
- With regards to *Rosetta energy score* differences, we observe that ProteinMPNN itself seems to generate very low energy structures and most CAPE-MPNN designs also fall within this peer group range or better. The energy scores of the designs are derived from predicted AlphaFold structures. Since AlphaFold incorporates an energy minimization step during prediction, the resulting structures are expected to at least reside in local energy minima of the force field used. This might not always be the case for experimentally determined structures. Consequently, designed structures may appear comparatively more stable based on a theoretical energy function than their experimentally determined counterparts. Some sequences exhibit highly unfavorable energy values. For instance, in the case of 1SQ3, even the PDB template file shows such unfavorable values. We speculate that this is because the ferritin, composed of multiple chains, achieves stability only within a fully assembled capsid-like arrangement. Generally, a Rosetta energy score per AA that exceeds the typical range of −3 to −1 points toward a failed design (See Assessment criteria).
- A striking feature of the *hydrophobic fitness* is that the designed proteins seem to be superior to naturally occurring ones. This might imply that ProteinMPNN has learned that hydrophobic AAs need to be buried within the protein’s core.
- One surprising feature of the packing density is how narrowly distributed the values are. Except for some outliers generated by e32b8ed0:epoch_20, the values are all quite close to each other.
- We also see favorable aggregation scores for the designed proteins (both average and maximum). This increases our confidence for the design of soluble proteins.
- The most difficult to interpret number is probably the pI. In general, the peer groups seem to have small ranges. We believe this is because the pI should be linked with location and functionality of the protein. We argue that ProteinMPNN itself is not trained to produce proteins with a certain function and location, just with a certain structure. We attribute the differences between the pI for data template structures and designs (including base ProteinMPNN) to the lack of consideration to function and location.

Overall, these results point to the necessity to assess each individual design independently. While most designs look very promising, in particular e32b8ed0:epoch_20 designs will need to be scrutinized.

### TEM-1 - Escherichia coli (pdb: 1M40)

As an example, we examine in detail the properties of designs for the 1M40 template. **The same information as for 1M40 is disclosed for all other specific and illustrative proteins in the *Appendix: Protein assessment details***. Figure 5 shows that the proposed designs are stable (see rosetta total, hydrophobic fitness, packing density) and mostly not expected to aggregate (see average and maximum aggrescan3d scores). However, we observe some significant difference in predicted isoelectric points. Nevertheless, that our method was able to reduce the number of predicted “strong” and “weak” binders within 1M40 can be see in Fig. 6. Finally, Box 1 depicts the template and designed sequences.

### Future work

While we have relied solely on computational methods to assess structure and visibility of the generated proteins, future work will have to use wetlab experimental methods to confirm this. X-Ray crystallography can be used to confirm structural validity. To confirm reduced immune-visibility, cells in humanized mouse models (with human MHC-I) could be induced to express the designed protein as well as the naturally occurring protein as a control using mRNA constructs (similar to recent COVID vaccines). Using their cells T-cell proliferation assays and ELISpot can be run to detect proliferation of activated memory cells.

ProteinMPNN has been used in various contexts. We restricted our evaluation to monomers and homo-oligomers. However, future work by experts in these particular fields of protein design should also look into the performance on hetero-complexes in particular protein–molecule and protein–DNA interactions. Also, like ProteinMPNN in general, our study is focused on the structure of proteins and does not directly take into account their function and or location within a cell.

## Conclusion

The renewed interest in protein design kindled by advances in ML has led to steady advances in this area. To use these *de novo* proteins as therapeutics in actual patients, prevention of unwanted immune-reactions is paramount. In this paper we analyze the potential of using DPO to tune the state-of-the-art protein design model ProteinMPNN to generate less MHC-I immune-visible proteins. We explore the trade-off between reducing visibility and quality of the generated designs. The findings point to the ability of DPO to effectively integrate a deimmunization goal into the ProteinMPNN protein design process. However, it also demonstrates its limitations, as visibility cannot be reduced to zero in the vast majority of cases and we see that structural similarity suffers for more extreme modifications. We also explored various indicators for protein properties and find that CAPE-MPNN designs’ values mostly fall into the range of experimentally determined peers of the template from the PDB. In particular, we observed very good hydrophobic fitness, packing density and aggregation scores. On the other hand, there seem to be quite large differences between the template proteins’ pIs and those of the designs. This is the case for the base ProteinMPNN as well as the CAPE-MPNN models. We think this has to do with the fact that these architectures are trained to generate sequences to structures without any information about functionality. This highlights the need to consider other biophysical features like pI in protein design foundation models such that they can correctly be taken into account in downstream applications.

## Supplementary Material

cape_mpnn_appendix_gzaf003

## Data Availability

Source code: https://github.com/hcgasser/CAPE_MPNN.

## Notes

**Editor:** Professor Timothy Whitehead
