# Supplementary material for: Tuning ProteinMPNN to reduce protein visibility via MHC Class I through direct preference optimization
Source: Protein Eng Des Sel. 2025 Mar 18;38:gzaf003. doi: 10.1093/protein/gzaf003 (PMC11970896; doi:10.1093/protein/gzaf003)
Supplement: cape_mpnn_appendix_gzaf003 [file cape_mpnn_appendix_gzaf003.pdf]

Appendix to: Tuning ProteinMPNN to reduce protein visibility via MHC Class I through direct preference optimization

Hans-Christof Gasser[*,1]
Diego Oyarzun[1,2]
Javier Alfaro[*,**,1,3,4,5]
Ajitha Rajan[*,**,1]

[1]School of Informatics, University of Edinburgh, Edinburgh, UK
[2]School of Biological Sciences, University of Edinburgh, Edinburgh, UK
[3]International Centre for Cancer Vaccine Science, University of Gdańsk, Gdańsk, Poland
[4]Department of Biochemistry and Microbiology, University of Victoria, Victoria, Canada
[5]The Canadian Association for Responsible AI in Medicine, Victoria, Canada
[*]Correspondence to: h.gasser@sms.ed.ac.uk, javier.alfaro@proteogenomics.ca, arajan@ed.ac.uk
[**]co-directed equally

March 2025

Acronyms

AA amino acid
Ab antibody
AR auto-regressive
CTL Cytotoxic T-lymphocyte
DPO Direct Preference Optimization
GAN Generative Adversarial Network
LLM large language model
LM language model
MD Molecular Dynamics
MHC-II MHC Class II
MHC-I MHC Class I
ML machine learning
MPNN message passing neural network
NLP Natural Language Processing
PDB Protein Data Bank
pI isoelectric point
PPO Proximal Policy Optimization
PWM position weight matrix
RBF radial basis function
REU Rosetta Energy Unit
RL reinforcement learning
RLHF reinforcement learning from human feedback
SOTA state of the art
TCR T-cell receptor
VAE Variational Autoencoder

A Appendix

A.1 Background on ProteinMPNN

Protein folding prediction is concerned with finding the 3D structure that an amino acid (AA) chain will fold into. After significant advances in this area - in particular by models like *AlphaFold* (Jumper et al. [2021], Abramson et al. [2024]) - the inverse problem, protein design, has also attracted renewed attention. Traditional approaches have been physics based like the *Rosetta Packer* (Leaver-Fay et al. [2011], Fleishman et al. [2011]). The current machine learning (ML) revolution is leading to a plethora of data-driven approaches using for example large language models (LLMs) (Ferruz et al. [2022], Madani et al. [2023]), Variational Autoencoders (VAEs) (Lyu et al. [2024]) and Generative Adversarial Networks (GANs) (Repecka et al. [2021]) to generate new amino-acid sequences. A particularly prominent model in this space is `ProteinMPNN` (Dauparas et al. [2022]). Although is a relatively small model (around 1.7m parameters) it supports protein complexes consisting of multiple chains, and parts of the produced sequence can be fixed upfront. This might be particularly interesting when the user has detailed knowledge of the desired functional mechanism and wants to ensure the necessary AAs are present.

A.1.1 `ProteinMPNN` data embeddings

The examples delivered by the above dataset are processed within the model to produce the following initial embedding tensors that are then fed through the encoder and decoder which are updating these.

Edge embeddings: The edge embeddings tensor will have shape $[B, S, N, D]$ (see main paper **Table 1**). The dimensions two and three determine which residue and neighbor the encoding in dimension four belongs to. Only during `ProteinMPNN` training, Normal random noise with standard variation of 0.2 Åis added to the coordinates of each residue. The nearest N neighbors (including itself) are then determined based on C_α distance between residues. Then the coordinates of the AA atoms are used to calculate inter atom distances. For each residue-neighbor pair, 25 inter atom distances are calculated. Each of these is between an atom (N, C_α, C, O and virtual C_β) of the residue and an atom of the nearest neighbor (N, C_α, C, O and virtual C_β). Distances are not encoded as a single numbers, but rather using 16 radial basis functions (RBFs) with centers spread between 2 and 22 Å(far distances - above 25 Å- will have values close to zero in all bins). So, at this stage each residue-neighbor edge will be encoded using 400 elements (25 x 16). These get concatenated with a relative positional embedding. This embedding can be one of 66 different learnt 16 element vectors. One of these vectors signifies that the neighbor is on a different AA chain, while the others signify that the neighbor is up to 32 positions before or after the residue concerned (further distances are clipped).

Overall the concatenated edge embedding tensor now has a shape of $[B, S, N, 416]$. This is reduced to $[B, S, N, D]$ by an affine transformation.

Node embeddings: These are a tensor of shape $[B, S, D]$. They start out being zero and are first updated by the encoder and afterwards by the decoder.

Token embeddings: These are only accessible by the *Sequence Decoder*. A sequence is represented as a list of AA indices (e.g. 0 is alanine, 1 is cysteine, ...). These are embedded into a D-dimensional space resulting in a token embeddings tensor of shape $[B, S, D]$.

A.1.2 `ProteinMPNN` information flow

Here we examine the information flow in a training forward pass. A sample step is the same for the encoder but the decoder needs to be called several times. This is a bit more complicated as it needs to be ensured that the decoder does not access information that is not yet available. For clarity, the following description leaves out normalization, dropout and activation steps.

Backbone Encoder

We now will discuss the *Backbone Encoder*. This is a message passing neural network (MPNN) consisting of a stack of three encoder layers. It receives the edge embedding, node embedding, nearest neighbor information as well as a mask that stores which residues have no known coordinates. The edge and node embeddings are updated by each layer of the stack.

Within a encoder layer, at first a message tensor is produced. This happens by combining the edge and node embeddings into a tensor of shape $[B, S, N, 3 \times D]$. For an edge between a residue specified in dimension 2 and one of its neighbors specified in dimension 3, its last dimension holds the following three embeddings (each D elements): the residue's node embedding, the edge embedding, the neighbor's node embedding. A sequence of three affine operations transforms this into the message tensor of shape $[B, S, N, D]$.

Elements in the message tensor that are linked to missing residues are first masked out, before summing over its third (neighbor) dimension. The result of this is then added to the node embeddings (skip connection). These updated node

embedding are then fed through a two layer fully connected network and the result added to the node embeddings again (skip connection).

To update the edge features, again a message tensor is produced as described above (using different parameters for the operations). This time, however, missing elements are not masked out. This message tensor is then added to the edge features (skip connection).

Sequence Decoder
A decoding order is sampled (missing residues are first). Based on this a backward and a forward mask are generated. An element in the backward mask is one if and only if a node's neighbor has already been decoded. The opposite holds for the forward mask.

The decoder receives the finally updated edge and node embeddings from the encoder as well as the masked sequence embeddings. With those the tensor EXV of shape $[B, S, N, 3 \times D]$ gets produced. Its last dimension hold first the encoder produced edge embeddings, then zeros and finally the neighbor's node embeddings (each D elements). In addition, the forward mask is applied to it (elements of neighbors that have already been decoded are set to zero).

Before the call of each of the three decoder layers, also another tensor ESV with a shape of $[B, S, N, 3 \times D]$ gets produced. Its last dimension holds initially the same elements at the beginning and end as EXV. Just the zeros in the middle are replaced by the AA embeddings of the neighbor. Also, the neighbor's node embeddings at the end get updated with every decoder layer call. Finally, the backward mask is applied to ESV (elements of neighbors that have not yet been decoded are set to zero).

Each decoder layer is then called with the node embeddings (by reference), and the sum of EXV and ESV. So the following embeddings are available for each edge.

- The residue's node embedding
- The edge embedding produced by the encoder
- The neighbor's AA embedding or zero if the neighbor has not yet been decoded
- The neighbor's node embeddings (encoder produced for not yet decoded neighbors)

The shape $[B, S, N, 4 \times D]$ tensor holding this per edge information is fed through three affine layers, resulting in a message tensor of shape $[B, S, N, D]$. The neighbor dimension of this message tensor is summed over and the result added to the node embeddings (skip connection). This updated node tensor is then fed through a two layer fully connected network and added to itself (skip connection). After this all node embeddings linked with unavailable residues are set to zero.

After the last decoder layer, there is an affine transformation that produces logits from the final node embeddings.

A.2 PWM presentation predictor example

Let us assume the peptide *SEELDKGEV* and our hypothetical patient (see main paper **Definition 1**).

Once, we need to construct the position weight matrix (PWM) for 9-mers corresponding to the hypothetical patient. For this we

1. Generate a million random 9-mers (5% change of each AA to be in each position)
2. Use *netMHCpan* (with a rank cutoff of 2%) to identify those that are predicted to be presented. We find that these are 19.6% of all random 9-mers
3. We construct the PWM by counting the number of AAs at each position within random presented 9-mers. This, divided by the number of random presented 9-mers is the probability of observing this AAs at that position. The log of this is recorded in the PWM
4. We calculate the the sum of log-probabilities for each 9-mer
5. The cut-off is then the 19.6% (see above) percentile of these log-probabilities. In our case this is -26.48

For this we first use *netMHCpan*

For the peptide *SEELDKGEV*, the probabilities for finding its AAs at their respective positions in presented peptides within PWM are: 6.3%, 1.8%, 3.4%, 4.2%, 4.2%, 5.0%, 2.8%, 6.3%, 10.3%. The logarithms of these are -2.76, -3.99, -3.38, -3.16, -3.17, -3.00, -3.57, -2.77 and -2.27. Their sum is -28.07 which is lower than the limit of -26.48 we calculated as cut-off (see above). We, therefore, predict that the peptide is not presented. If in contrast the second position in the peptide had been a proline, which was observed in 9.5% of all presented peptides, then the sum of the log-probabilities would have been -26.43, which would be above the cutoff point and therefore predicted to be presented.

	position								
	1	2	3	4	5	6	7	8	9
A	-2.92	-2.75	-2.89	-3.00	-3.04	-3.04	-3.09	-2.82	-3.20
C	-3.72	-4.63	-3.52	-3.72	-3.59	-3.54	-3.47	-3.32	-3.69
D	-3.48	-6.30	-2.83	-2.65	-3.17	-3.31	-3.42	-3.59	-9.55
E	-3.34	-3.99	-3.38	-2.68	-3.10	-3.20	-3.09	-2.77	-6.18
F	-2.65	-2.87	-2.76	-3.20	-3.01	-2.92	-2.95	-3.07	-1.87
G	-3.26	-3.95	-3.41	-2.85	-2.91	-2.99	-3.57	-3.30	-6.20
H	-2.75	-2.19	-2.90	-3.03	-2.77	-2.83	-2.67	-2.89	-3.89
I	-2.89	-3.46	-2.91	-3.23	-2.93	-2.81	-2.88	-3.11	-2.31
K	-2.83	-3.29	-3.05	-2.93	-2.84	-3.00	-3.18	-2.89	-4.93
L	-3.12	-2.85	-3.03	-3.16	-3.06	-2.99	-2.87	-2.96	-1.88
M	-2.86	-2.92	-2.77	-3.24	-3.19	-3.09	-2.90	-3.07	-2.06
N	-3.02	-4.36	-2.91	-2.95	-2.96	-3.03	-3.01	-3.09	-8.45
P	-4.09	-2.35	-2.91	-2.52	-2.74	-2.71	-2.88	-3.17	-4.89
Q	-3.06	-2.90	-3.04	-2.90	-3.02	-2.94	-2.85	-2.78	-5.89
R	-2.77	-2.33	-3.14	-3.12	-2.88	-2.97	-2.98	-3.02	-5.14
S	-2.76	-3.02	-2.96	-2.87	-2.99	-2.93	-3.02	-2.72	-5.16
T	-2.92	-3.16	-3.13	-3.00	-3.00	-2.85	-2.89	-2.66	-4.12
V	-2.89	-3.30	-3.13	-3.21	-2.96	-2.85	-2.86	-2.86	-2.27
W	-3.23	-2.82	-2.97	-3.11	-3.02	-3.18	-2.90	-3.53	-2.24
Y	-2.54	-2.41	-2.70	-3.19	-3.03	-3.05	-2.89	-2.88	-2.11

Table A1: **PWM for presented 9-mers in our hypothetical patient:** Each column corresponds to a position in the 9-mer and each row to one of the 20 standard AAs. The values are the log-probabilities of finding the corresponding AA at that position in a presented 9-mer

A.3 Protein assessment details

Figures A1, A2 show the information for the specific validation proteins corresponding to the **Figures 3, 4** in the main paper. Then follow pages with detailed information about the generated designs for specific validation, specific test and illustrative proteins (mixed and ordered alphabetically).

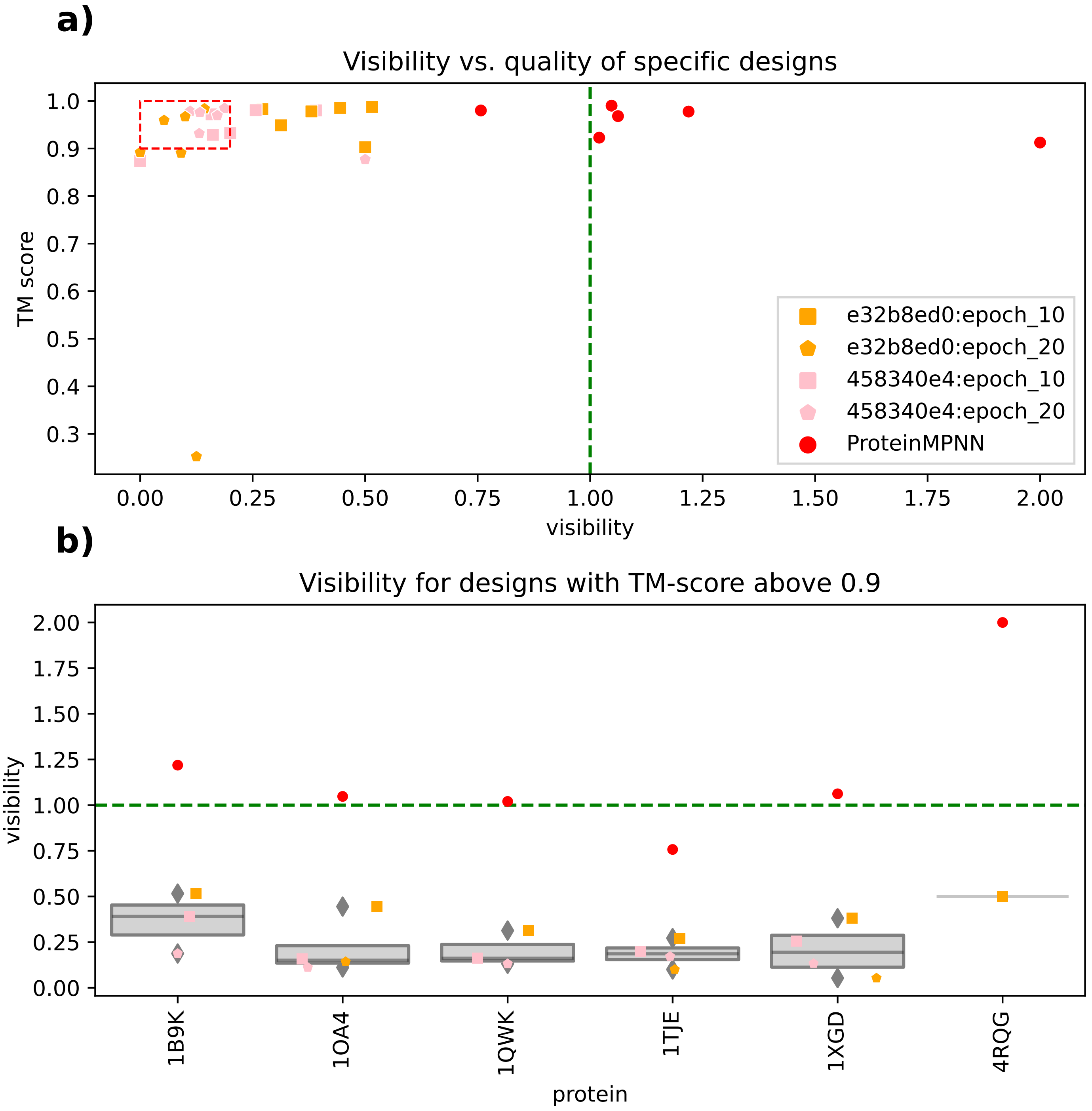

Figure A1: **A broad performance range:** This figure looks into specific valuation designs. In plot **a)** the quality of generated specific designs (as measured by TM-score on the y-axis) gets compared to the relative visibility (x-axis) for two Direct Preference Optimization (DPO) hyper-parameter selections after two epochs of tuning. Each point represents a design for a specific backbone using the checkpoint. As for the specific test designs (main paper **Figure 3**), We find that most designs have high TM-scores above 0.9. Not all of them are less visible than the original sequence (visibility < 1.0) and we also see that the `ProteinMPNN` designs in red tend to have far higher visibility. Furthermore, some designs also only show a maximum of 20% the number of visible peptides in the designs (red dashed box). In plot **b)** we then look into the distribution of visibilities of designs for specific backbones. These are subset of designs from plot a) that satisfy the condition that their TM-score is above 0.9. The dashed green lines represent the visibility of the PDB proteins.

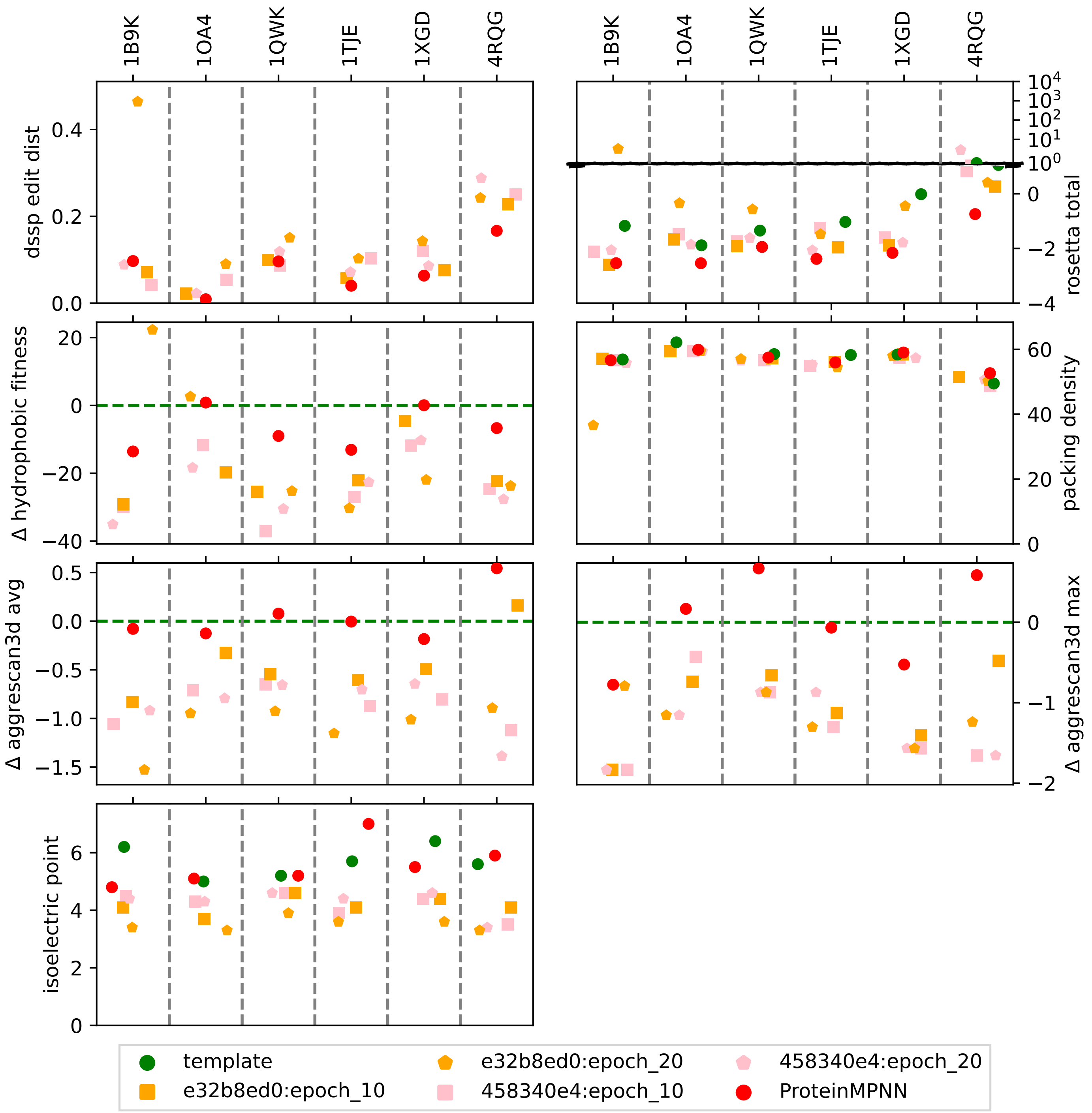

Figure A2: **Quality indicators for designs:** The figures above show the distribution of quality indicators for the `ProteinMPNN` and `CAPE-MPNN` designs by specific validation set protein template. Each dot signifies a design. The green bars in the background depict the range of values for identified naturally occurring peer proteins (only included if at least five were identified). For all indicators except packing density and isoelectric point (pI), lower values are considered favourable. With regards to pI, we want to be as close as possible to the template protein and with regards to packing density, higher values are considered favourable (Stam and Wood [2021]). The figure showing "rosetta total" is split in two parts. The bottom part (range -4 to 1) depicts the normal range of values. To also depict the outliers that have problematic values we also show a second log scale above.

A.4 1A3H

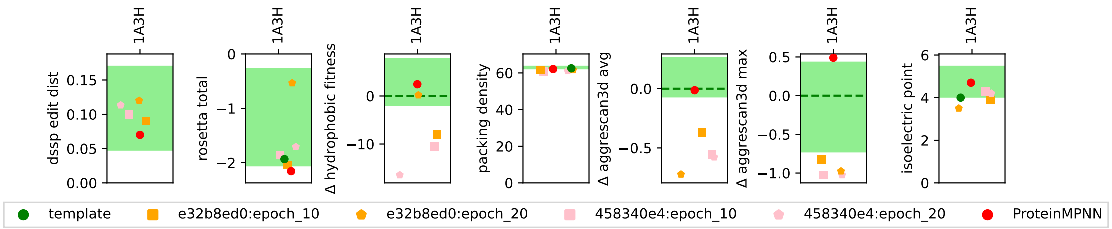

Figure A3: Focus on 1A3H

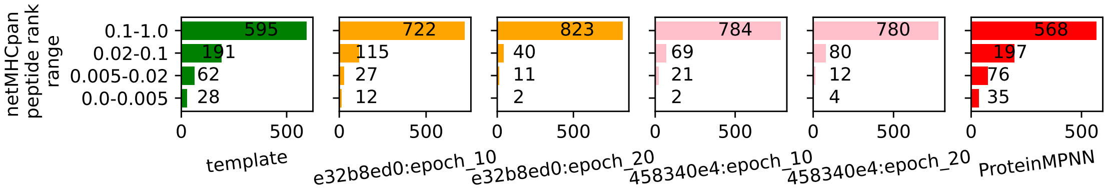

Figure A4: netMHCpan ranks for 1A3H designs

template

SVVEEHGQLS ISNGELVNER GEQVQLKGMS SHGLQWYGQF VNYESMKWLR DDWGINVFRA AMYTSSGGYI DDPSVKEKVK
EAVEAAIDLD IYVIIDWHIL SDNDPNIYKE EAKDFFDEMS ELYGDYPNVI YEIANEPNGS DVTWGNQIKP YAEEVIPIIR
NNDPNNIIIV GTGTWSQDVH HAADNQLADP NVMYAFHFYA GTHGQNLRDQ VDYALDQGAA IFVSEWGTSA ATGDGGVFLD
EAQVWIDFMD ERNLSWANWS LTHKDESSAA LMPGANPTGG WTEAELSPSG TFVREKIRES

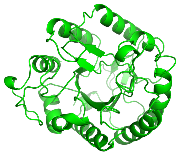

e32b8ed0:epoch_10 - TM score: 0.993, Seq Recovery: 0.51, Visibility: 0.43

DFVTKNGKLS IKDGKLVNEN GEEVVLKGVS SDNLQDNGDY VNDSNIKDLK DNWGINIYRA NVDRDNGGLV DDPSILDKAY
DAIDACEKND IYGVFDLHNG NTGNPNDNID LDKEVMKEIA EKYGDKDNVI YDINNEPNGA DVTWDTDIKP YADEVIPVIK
ENDKDNIIIV GTANNSTDVV SAADNKLDDP NVMYAFHAAA GEELDKKMED VKKAQDKGAA IFMSEWTTAN WNGDGGVHLD
EAQKLWDFMD ENNISGTYWA DSNDNRDSAL RTSGASPDGG WSDSQLSESG QAVAKRIRDS

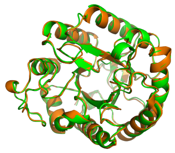

e32b8ed0:epoch_20 - TM score: 0.979, Seq Recovery: 0.38, Visibility: 0.14

DDVDDNGKLS VKDGKEVDEN GNETTLKGNS SDDNNKDGDY DNKDNIDDDK NEDGANIWGA NNNVDGGGRD DDPSTKDKDD
DSNDACEDNN IYGDSNNNNG NDGNPNDNKD KDKEDMDEKA KKYGDKDNVI YDIDNEPNGD DVTWDDDIKP NADEVIPVIK
ENDDDNIIDV GTANNSTDVD TAADNKLDDD NTRANNHSSA GNDGDDDKKN VDKAQDKGEA VNNGEWNGAN DNGDGGVNDD
EDDKENDYND DNGISSNYWS DSNNDDDASD RTKDASPDGG DTDDDDSENG KKRKDDIKDA

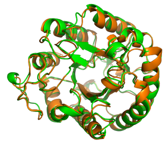

458340e4:epoch_10 - TM score: 0.989, Seq Recovery: 0.46, Visibility: 0.26

DVVKKNGKLS IKDGKIVNEK GEEIVLKGIS SDNLQKDGEY VNCKNIKDKK DNWGINIWRA NCNIGNGGIK DDPSILDIGN
KGIDCCEKED IYCVFDNNNG TDGNCNTDID LTKEINKEIA EKYGDKDNVI YDICNEPNGK DVTWKDNIKP YADEVIPVIK
ENDKDNIIIV GTANNSTDVV EAAKNKLDDK NVCYACHWCA GEKGEQKKKD VKKAQDEGAC IWCSEWSTAK GNGDGGVNEE
EADEWWDFCD ENNISCCYCC DSNEDKDAAL RTKDANPDGG WKDEELSESG KKVKDKIKKS

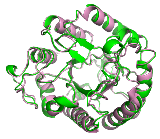

458340e4:epoch_20 - TM score: 0.989, Seq Recovery: 0.45, Visibility: 0.18

DFVKKNGKLS IKDGKIVNKD GEEVVLCGIC SDGLQKNGEY VNCKSIKWCK DECGCNIWRC NCKWGNGGWI DDPSIKDICD
KGIDCCEEND IYCVFDCCNG DIGNPNDNID KIKECMKEIA EKYGDKDNVI WDICNEPNGK DVTWEDNIKP YADEVIPVIK
ENDKDNIIIV GTGNNSTDVV EAAKNKLDDK NVCYACHFCA GEKGDEKKKD VKKAQDEGAC IFCSEWSCAK GNGDGGCNFD
EFDKWCDFCD ENNISWCYCC WSNDDKDNAC LTKNASPDGG WKDEELSDCG KKVCDIIKKK

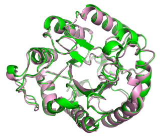

v_48_020 - TM score: 0.993, Seq Recovery: 0.53, Visibility: 1.23

MFVEKNGKLS IKNGKLVNEN GEPVVLKGFS SDALQKYGEY LNYSSMKWLK DEVGINIFRA NMNMDSGGYI EDPSIYDLVD
KAIDAAEELD IYAVVDAHNW SLYNPNTYLD ERIELFEKIA KKYGDKPHVI YEIANEPSGA DVTWEKDIKP YAEKVIPVIR
KHNPDNIIIV GTPNWSRDVV TAANNPLAYP NVMYAFHFAA GEPLEEHMKN VDKALAKGAA IYCSEFSLAK WNGDGGVFPE
QFNKMWDYFD EHNISYTYWS LSTANRDAAA LTSTASPDGG WTDSQLSAAG QLVKAKIRAA

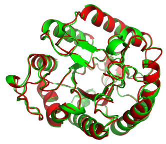

Box 1: **Template and designed sequences for 1A3H**

A.5 1B9K

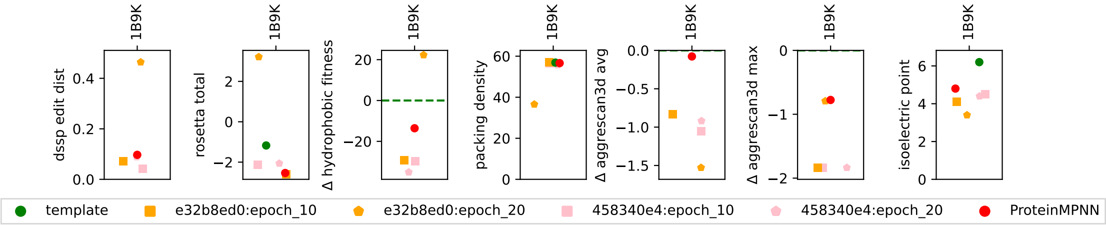

Figure A5: Focus on 1B9K

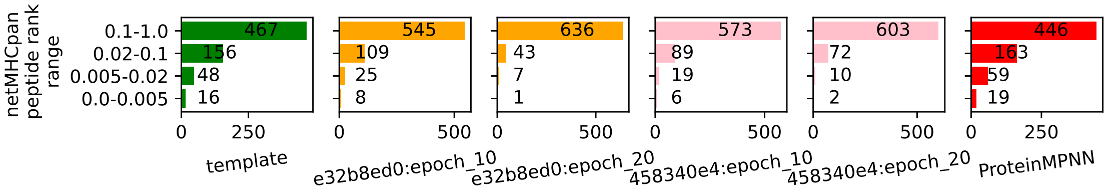

Figure A6: netMHCpan ranks for 1B9K designs

template
EDNFARFVCK NNGVLFENQL LQIGLKSEFR QNLGRMFIFY GNKTSTQFLN FTPTLICADD LQTNLNLQTK PVDPTVDGGA
QVQQVINIEC ISDFTEAPVL NIQFRYGGTF QNVSVKLPIT LNKFFQPTEM ASQDFFQRWK QLSNPQQEVQ NIFKAKHPMD
TEITKAKIIG FGSALLEEVD PNPANFVGAG IIHTKTTQIG CLLRLEPNLQ AQMYRLTLRT SKDTVSQRLC ELLSEQF

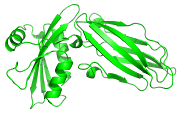

e32b8ed0:epoch_10 - TM score: 0.987, Seq Recovery: 0.33, Visibility: 0.52
GDNFEDFINK DSGTLYKDDI IEINIDKTYD GNTGTIKLTF KNLTDEDITD FDPKIEQSED LKEKLDIKYD DGDKTIAGGE
EKDLTIDVTA KAPFKDKPTL KITYTYNGEE KEDEVKLPIV LNDFAEPVEM NKEEFEEEKN KVTDPDNIKT TTKKRKNPND
KEKIKEKIKG FGFALLEDVD DDKNNFVGAG TIKTEDKDIK VLLELKPDEE NDSDTLTVKA DDAEVADIVN EELSKLF

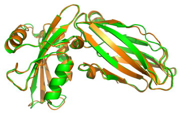

e32b8ed0:epoch_20 - TM score: 0.252, Seq Recovery: 0.22, Visibility: 0.12
GDNTDDFIDK DDGTLDEDDD TKVDQDGDYD GNKGTVKLTT TNKTDDDADD DDDTVDQDDD LKDKLDIDED DGDDTIDGGD
SEDTTLDTDN KDNYDDTPTL DRDYDLDGDD KSDSSDLPIN NNKNKEAEEE DKDEWEKKKN EAEDDDDIEE ETSDNKNDND
DDEIKEKEKG FGDAVLEDVD DDDNNYDGKG TIKSEDKDDD VQSKLENDEE NDKDTLTVIA DDAETAKTEN EDKKKLY

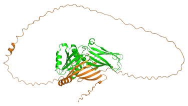

458340e4:epoch_10 - TM score: 0.980, Seq Recovery: 0.33, Visibility: 0.39
GNNFENFINK KNGTLYKDDI IEINIDKECD GNKGTIKIKI KNKTDEDIKN CDCKIECSED LKEKLKIEYD EGDKTIKGGE
EKDITIKVTC KKPFKEKPIL KIKYEYNGKE IEKEIKLPIV LNDFMEPKEM NKEEWEEEWD KIEDEDEIKT ITKKRKNKND
KEKIKEKIKG FGGALLEDVD DDKNNFVGNG IIKCEEKDID VKFKLKPDEE NDTDTLTIKA DDEEVADILC EELSKLF

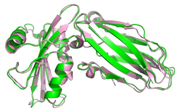

458340e4:epoch_20 - TM score: 0.985, Seq Recovery: 0.30, Visibility: 0.19
GDNYEDFICK KDGTLFENDI FKVDIEKTCE GNKGTIKITF TNKTDKDCTN FDPKIECDDE LKEKLDITSD DCDKTIEGGK
SIDKTINVTC KDIFSGKPIL KIKCEYNGED KEYEIKLPIN INDFCEPKEC DKETFWEECN KCKDKDCICE KTCKRKNDND
KEKIKEKIKG FGFACLEDVC DDKNNFCCCG IIKCENKDIK CQCIIKPDEE NNTCTITCIC DDSECCKICC EEISKLF

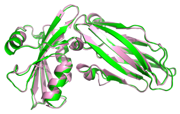

v_48_020 - TM score: 0.978, Seq Recovery: 0.39, Visibility: 1.22
GDNFEEFINK KSGVLYENDI IKVTISATYS GNKGTITLTF TNLTDVPLTN FTPTIVLSPE LQKKLTITYA PVDPTLAGGS
SVTLTLNVTA KDPFSERPTL VITYTYKGKE HKESITLPIV LNDFLEPKPM DKETFWALYN SVTDPENVVE ITFKAKNPFD
RETIKAKLKG FGFALLENVD PDPNNFVGAG IVKFENKDIK VLLILKPDLE KNTFTLTIIA DDPEVAKILA ELLSKLF

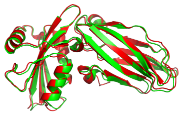

Box 2: **Template and designed sequences for 1B9K**

A.6 1OA4

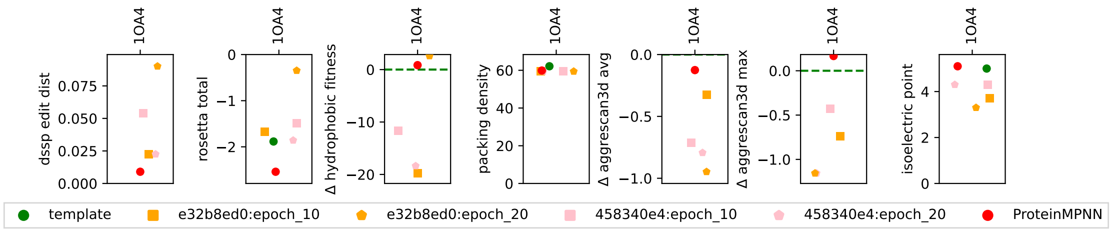

Figure A7: Focus on 1OA4

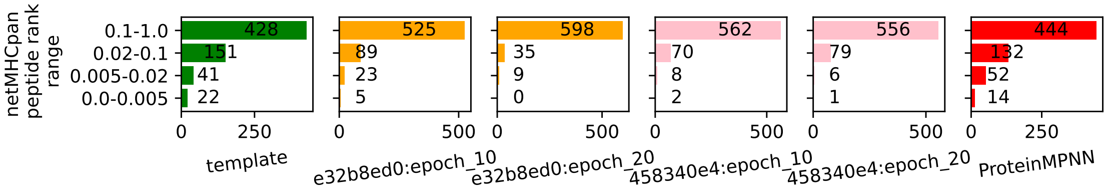

Figure A8: netMHCpan ranks for 1OA4 designs

template

NQQICDRYGT TTIQDRYVVQ NNRWGTSATQ CINVTGNGFE ITQADGSVPT NGAPKSYPSV YDGCHYGNCA PRTTLPMRIS

SIGSAPSSVS YRYTGNGVYN AAYDIWLDPT PRTNGVNRTE IMIWFNRVGP VQPIGSPVGT AHVGGRSWEV WTGSNGSNDV

ISFLAPSAIS SWSFDVKDFV DQAVSHGLAT PDWYLTSIQA GFEPWEGGTG LAVNSFSSAV NA

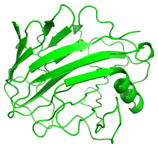

e32b8ed0:epoch_10 - TM score: 0.985, Seq Recovery: 0.50, Visibility: 0.44

TTTITEKDGS TTIGNKFVAS NDNWGTTDTT AIEVGANGFT ITKSDGSNST DGNSKGNPHI FDGCENGVCA PGTTLPQKIK

DIGSAPASVS ITTTDTGVYD AEGDIDIDKT AKTSGTADTE IDIIFDRNGD VKPVGEKIGK ATVNGKNWDV YEGSDGTRNI

LSLVADSPIA SESFDVKDFV DLAIDKGKAT NDDYLTDIGW GFDNWDGGEG NAVNDFSASV NA

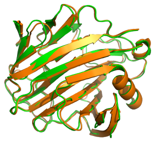

e32b8ed0:epoch_20 - TM score: 0.984, Seq Recovery: 0.42, Visibility: 0.14

TETICDKDGS TTIKDKYVAD NDNDGSDDDE CIDVDDNGFT ITKNDGSNDT NGDGKGDPHI YDGCEDGNCA PGTNLPQKIK

DIGNAKKGGK YTKTDDGTYD AEDDIDIDAT PKTSGTDDTE IDNVDSDNGD VDDVGEEIGD DEINGEDYDV WEGDDGNKDI

LGNDADDDID SKDYDAKDDV DKAIDKGKAT DDDYLNNIGS GFDNYDGGEG NSVDDFSADV DD

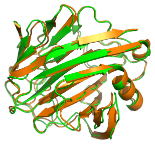

458340e4:epoch_10 - TM score: 0.972, Seq Recovery: 0.43, Visibility: 0.16

KKIICEKDGS TTIKDKFVVE NNNWGTDDEC CIEVGENGFK IVKCDGSNST DGNSKGDPHI FDGCENGKCA KGTTLPKKIK

DIGKCPTSVK IKTTDDGVYD VEDDIDIDKT AKTDGTTDTD IDIIRDKNGD VKPIGKKIGK ETINGKNWDV WEGNDGKKNI

LSLVADEKID SESKDVKDYV KLAIDKGKAT EDDYLCDIES GFDCWEGGEG REIKDFSAKC ED

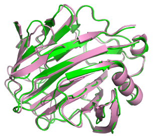

458340e4:epoch_20 - TM score: 0.978, Seq Recovery: 0.44, Visibility: 0.11

DKTICEKDGT TTIKDKFVVS NDNWGTDDEC CITVGENGFK ITKSDGSTST DGDCKGDPHI YCGCDDGICA PGTNLPKKIK

DIGKCPCSVE FKFTDDGVYT AKYDIDIDKE PKTDGITDTE IEIVFDKNGD VKPVGKEIGE AEINGKKWKV YEGSDGKRDI

LSLVAKEKIE KDSFDIKDCI KLCIDKGKAT EDDYLCRIGC GFCNWKGGEG NEVKDFDCKC EE

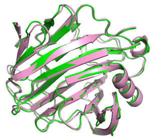

v_48_020 - TM score: 0.990, Seq Recovery: 0.52, Visibility: 1.05

MKVLSEKNGY TYIKNKYVVA NNNWGTDAPV AIEVGENGFT IIKSEGSNPV TGAPKGFPHI YYGCINGVCA PGTTLPMKIK

DIGKAPVSAS FTYTDTGVYT AQWDILIDKT PKTSGTASTE IEVIFARNGP VKPVGKYVGE AEVNGEKWEV WIGSDGTRNI

LSLVAKEPIA SKSLDAKDFL DLAIEKGLAT PDDYLLSISA GFAPWEGGVG LSVKSFSAEV LK

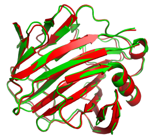

Box 3: **Template and designed sequences for 1OA4**

A.7 1P3C

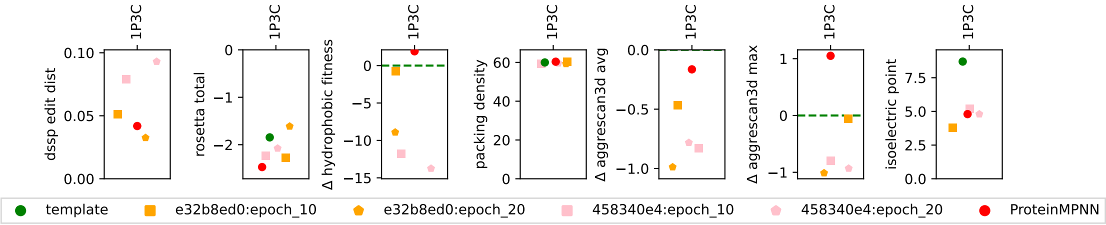

Figure A9: Focus on 1P3C

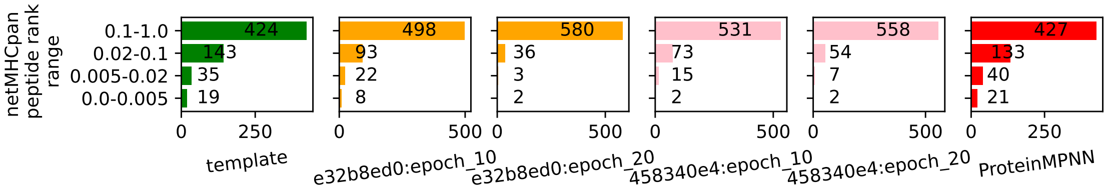

Figure A10: netMHCpan ranks for 1P3C designs

```
template
VVIGDDGRTK  VANTRVAPYN  SIAYITFGGS  SCTGTLIAPN  KILTNGHCVY  NTASRSYSAK  GSVYPGMNDS  TAVNGSANMT
EFYVPSGYIN  TGASQYDFAV  IKTDTNIGNT  VGYRSIRQVT  NLTGTTIKIS  GYPGDKMRST  GKVSQWEMSG  SVTREDTNLA
YYTIDTFSGN  SGSAMLDQNQ  QIVGVHNAGY  SNGTINGGPK  ATAAFVEFIN  YAKAQ
```

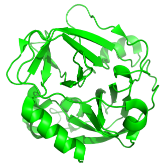

```
e32b8ed0:epoch_10  - TM score:  0.992, Seq Recovery:  0.57, Visibility:  0.56
NIIGDDKRTE  VTDTTVAPYN  AIAYVTFDNG  YCTGTLIDNN  KVLTNAHCVY  NTNTNTYAGK  GTVTPGQDNS  TAVNGSANET
DYYVPDGYIN  TGDDKYDYAV  IDTDTNIGDT  VGKLDIKEVD  TLKGENVTVA  GYPGNNITAT  GKVSQWEESG  KVTDEDEDLM
YSDIDTDGGD  SGAPFLDDDN  KIVGIHSGGA  DGGTKNYGPK  ANKEVKDFID  EAKKK
```

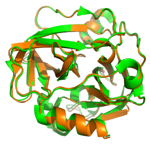

```
e32b8ed0:epoch_20  - TM score:  0.989, Seq Recovery:  0.45, Visibility:  0.09
NIIGDDKRKE  VDDTTTDPNN  SIADVTDGDG  SGTGTIIDDN  KILTNAHDDY  NNNNNKNADK  GKSTPGEDNS  EAKNGESNET
DDDIPDGAKD  TGDDSDDYSV  RDTDKDIGKD  VGKKDTKEVD  TLKGEDVTVT  GNPNDEKDKT  GKNSQYEESG  KVTDEDDDNA
YSDIDTDGGD  SGADFLDKDN  KIVGVNSGGA  NNGTKNGGPK  ANDDFKKDID  DAKKK
```

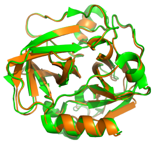

```
458340e4:epoch_10  - TM score:  0.989, Seq Recovery:  0.46, Visibility:  0.31
NIIGEDKRKE  VEDTTKKPNN  CICYVTFDDG  YCTGTLIDDN  KILTNAHCVY  NKETNKYAGK  GTATPGIDNS  EAKNGSSKIT
DYDIPEGYKN  TGDDKWDIAC  IKLDKDIGKK  VGKLDIKEVD  KLKGEKVKLC  GYPGNNIDKT  GKVSQWCEEG  EITDEDEDLM
YSDIDTDGGD  SGAPFIDKND  KIVGIHTGGA  KGGTKNYGIK  ANKKVKDFID  KCKKK
```

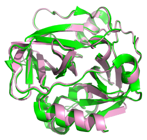

```
458340e4:epoch_20  - TM score:  0.989, Seq Recovery:  0.45, Visibility:  0.17
NIIGEDKRKK  VEDTTKKPYN  CICYVTFDDG  NCTGTLIDDN  KILTNCHCVW  NCETNKWAGK  GTVTPGQNDS  EAKNGTTKET
DYCVPEECKN  TCDDKWDIAC  IKTDKDIGKK  VGKLDIKEVD  TVKGEKVKVC  GYPNNNIDKT  GKVSQWCEEG  EITDEDEDLM
YTDINTDGGC  SGAPFLDKDD  KIVGINTGGA  KNGTKNYGIK  CNKKVKDFID  ECKKK
```

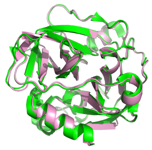

```
v_48_020  - TM score:  0.992, Seq Recovery:  0.52, Visibility:  1.13
AIIGEDKRTE  VTDTTVFPYN  AIAYVTFSSG  FCTGTLIADN  KVLTNAHCVY  NYNTKTYAGL  GTVYPGMDNS  TPLNGSAKMT
AYYIPQEYID  TGSWKYDYAV  IDLDTDIGKK  VGYLEIKEVD  TLKGETVTLA  GYPGNYIAAT  GKVSLYEETG  KIVSEDENLM
YSKIDTDGGN  SGAPFLDSNN  KIVGIHTGSA  KGGTINYGPK  MTKEAKKFIE  EALKK
```

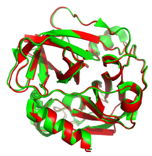

Box 4: **Template and designed sequences for 1P3C**

A.8 1PGS

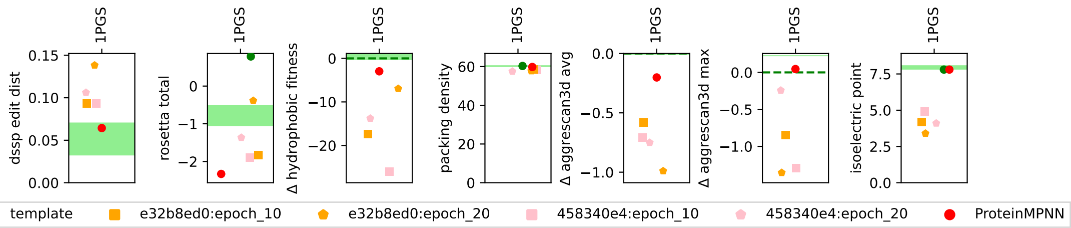

Figure A11: Focus on 1PGS

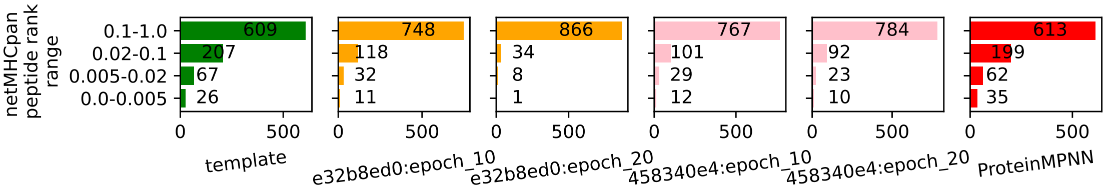

Figure A12: netMHCpan ranks for 1PGS designs

template
DNTVNIKTFD KVKNAFGDGL SQSAEGTFTF PADVTTVKTI KMFIKNECPN KTCDEWDRYA NVYVKNKTTG EWYEIGRFIT
PYWVGTEKLP RGLEIDVTDF KSLLSGNTEL KIYTETWLAK GREYSVDFDI VYGTPDYKYS AVVPVIQYNK SSIDGVPYGK
AHTLGLKKNI QLPTNTEKAY LRTTISGWGH AKPYDAGSRG CAEWCFRTHT IAINNANTFQ HQLGALGCSA NPINNQSPGN
WTPDRAGWCP GMAVPTRIDV LNNSLTGSTF SYEYKFQSWT NNGTNGDAFY AISSFVIAKS NTPISAPVVT N

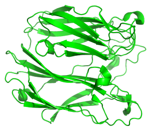

e32b8ed0:epoch_10 - TM score: 0.985, Seq Recovery: 0.49, Visibility: 0.46
EETETINAFE NAKTSSGNGG SKSAEGTFKF PEDTTKVVNI DMHIKEDCPN NSCDPNCNYC KVEVKDKETG KWLTIAKWIT
PNNVGTEELK DGLKSDVTDA KDLIKGDTEL KISCDVDNSK GRTFSTSFDI TYGDPEYKYS EIEEVIDKDE SENDGIPYGT
DNDIKTEKKV TLPANTEKAY LKVQTYGSGD ATPTAAGNKG AAEDRETTDT IEVDGKPTFS INQGGLGCAN NPINNQSPGN
YTDDHAGWCC GQVVPTTTFD LDDSLKGKTF TIKYKQEPYT NDGTNGDAYK SVTIDVVAES NTKIDKPKVT D

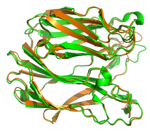

e32b8ed0:epoch_20 - TM score: 0.969, Seq Recovery: 0.38, Visibility: 0.10
MTTTTNNGFD NAKCSDGNGG SKSDENTNDY PDDTTDTDDI DQNTKENCDN NNCDGNCNDC KKEVKDDDDG DWKTISEWRS
NNNNGTENDE NGLKDNVTDD KDLIKGDVDE KISCDTDNSN GRTYSTSDDI TEGDPDNKYS ETEDIIDNDD GDDDGIDVGD
DDDDDTKKNV TTDDNTDDAD LNNKKSGSGD ATPNDAGDKG AAEDKDDTDT IKVDGEDTFD DDDGGIGCAN NKNNNGSPGN
DNDDHAGWCC GETVPKTTNK LDDDKKGKTF TVESEQEDYT NDGTNGDADD DESTDIVDKS NTEIDKPEVT D

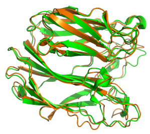

458340e4:epoch_10 - TM score: 0.990, Seq Recovery: 0.49, Visibility: 0.44
EETKKIDCFK DVKCSDGNGG SKSAEGTFKF PKDTTKVVKI DMNIKEDCPN NSCDPNCNYC KVEVKDKETG KWLTIAKWIT
PNNVGTEEIK DGLKKDVTDF KDLIKGDVKL KITCDVDNEK GRTFTISFDI TYGDPEYKYS EIEEVIDKDE GDNDGIPYGK
DNDIKTKKKV TLPKNTEKAY LKIQIKGSGD ANPKDAGNKG AAEDRKTTDT IKIDGKDTFK IDKGGLGCDK NKINKQDPGD
WKKDKAGWCC GEVVPTTTCE LDKELIGKTF TIEYKFEDYT NDGTNGDAYK SITINVVCES NVKIEKPKVT D

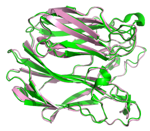

458340e4:epoch_20 - TM score: 0.980, Seq Recovery: 0.48, Visibility: 0.35
DEVKKIDCFK DVKCSDGNGG SKCAEGTFKF PEDTTNVVKI DCYIKEDCPN NTCDPNCDYC KVKVKDKETG KWLTICKWIT
PNNVGTEELK DGLKCDVTCC KDLICGETKL KICCDVDNEK GRTFSISFDI TYGDPEYKYS RIVEVIDYDE DDDDGIDYGV
DDDKKLKKKV TLPKNTEKAY LKIIIKGDGD ACPKDEGNKG CCEDCERTDK IKINGKETFD IDMGGLGCEN NPINNCEPGD
CKDDKAGWCC GQVIPEKICE LDKELIGKTF TIEYEFEDRE NDGTCGDCDV CITIDVVCES NVEIEKPKVT D

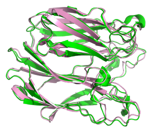

v_48_020 - TM score: 0.994, Seq Recovery: 0.56, Visibility: 1.04
MKTITINAFQ NVKTASGNGQ SKSAEGTFNF PEDTTNVVKI DMYVKESCPN NSCDPNSRYC YIQVKDKETG EWLLIAAWIS
PYNVGTEELP NGLKTDVTDF KELLKGETEL KIYCEVTNSK GRTFSVSFDI TYGTPEYKYS RIVPVIQYDE SSTKGIPYGV
PHNILLKRKV TLPKNTEKAY LIVRAYGYGN ASPYAPGNRG AAEWRPRTDT ILINGKPTFS LPHGALGCAK NPINKQAPGN
WKPDRAGWCP GEPVPTHRFE LPSSLFGKTF TIEFKLEPYT NDGSNGTAYI ALSIYVVVES NVPIEKPVVT D

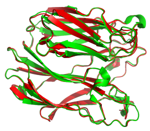

Box 5: **Template and designed sequences for 1PGS**

A.9 1QKD

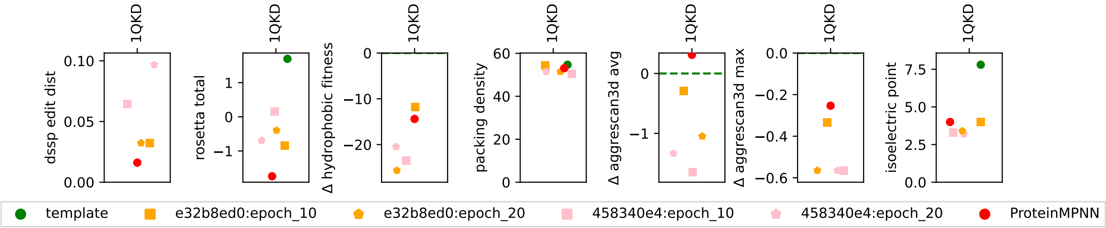

Figure A13: Focus on 1QKD

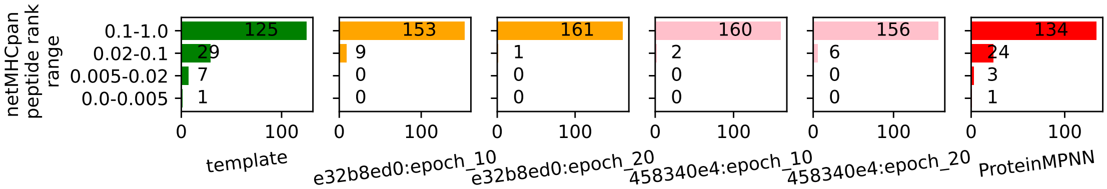

Figure A14: netMHCpan ranks for 1QKD designs

template
RICFNHQSSQ PQTTKTCSPG ESSCYNKQWS DFRGTIIERG CGCPTVKPGI KLSCCESEVC NN

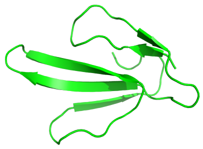

e32b8ed0:epoch_10 - TM score: 0.903, Seq Recovery: 0.46, Visibility: 0.00
ITCYNNNGND PDTTTTCASG VNWCYKKTSS DSSGEVTERG CGCPSVESGV ENCCCNSDNC NK

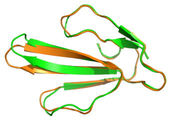

e32b8ed0:epoch_20 - TM score: 0.907, Seq Recovery: 0.41, Visibility: 0.00
KTCNNNKGDD ATTTTTCADG ETDCYKKTSD DDDGTKTDTG CGCPDVDDGE NNCCCDCDNC NG

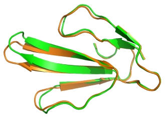

458340e4:epoch_10 - TM score: 0.911, Seq Recovery: 0.38, Visibility: 0.00
KTCNDEDGDD EEETTDCADG ETECYKIEWD DEDGEEEERG CGCPEVEDGE ENCCCNCDKC NK

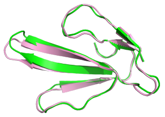

458340e4:epoch_20 - TM score: 0.934, Seq Recovery: 0.38, Visibility: 0.00
LTCNDEDGDD EDETTDCAEG EKWCYKKTWD DEDGEEVERG CGCPECEEGV DCCCCNCDNC NE

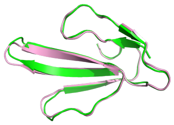

v_48_020 - TM score: 0.932, Seq Recovery: 0.46, Visibility: 0.50
LTCYSNSGNA PPTTTCPSG ETYCYRITWT DSNGTVVERG CGCPSVPSGV NLCCCNCDLC NQ

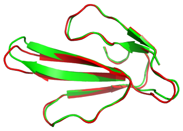

Box 6: **Template and designed sequences for 1QKD**

A.10 1QWK

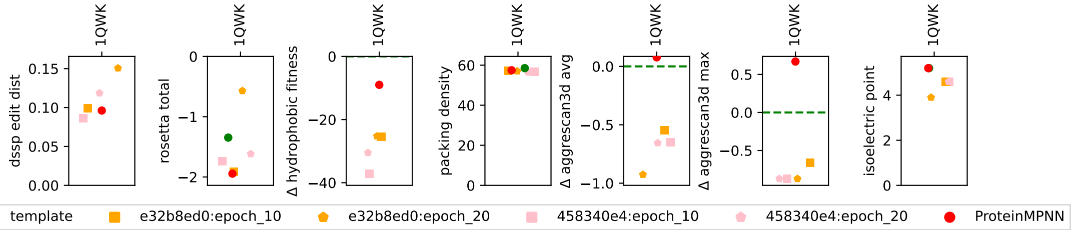

Figure A15: Focus on 1QWK

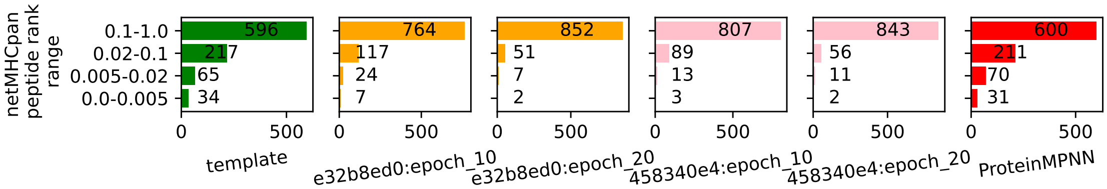

Figure A16: netMHCpan ranks for 1QWK designs

template
TASIKLSNGV EMPVIGLGTW QSSPAEVITA VKTAVKAGYR LIDTASVYQN EEAIGTAIKE LLEEGVVKRE ELFITTKAWT
HELAPGKLEG GLRESLKKLQ LEYVDLYLAH MPAAFNDDMS EHIASPVEDV WRQFDAVYKA GLAKAVGVSN WNNDQISRAL
ALGLTPVHNS QVELHLYFPQ HDHVDFCKKH NISVTSYATL GSPGRVNFTL PTGQKLDWAP APSDLQDQNV LALAEKTHKT
PAQVLLRYAL DRGCAILPKS IQENRIKENF EVFDFSLTEE DIAKLEESKN SQRLFLQDFM TGHPEDAFAA ER

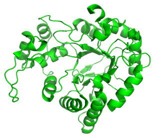

e32b8ed0:epoch_10 - TM score: 0.949, Seq Recovery: 0.42, Visibility: 0.31
MKTITLSNGK KLPILMLGTG GATPEEAKEA VKTAIKNGIK GIDTAEGNGN EKAVGEAIKE AEEEGIVKRE DLNIITKIGP
AGLRPGKARE AVEKSLEELG RDKADVVLAG NTCATNDDGT EFVDAPVTEI HKELDEIKKE GLADAIGVSN ANNDQIDEAE
ATGGTPIDVW KDEINIDYPN KDLIAHCKER NIGVIADKPL GKKGKEENVD EDGTKGESTD KESQQTDENV KKLAKEKGKT
PEQICIKYCI DKGCAVTPGA KDKKDIEENG KVFDFSLTEE EIKELEKSGK NERQDKREET KGHPNDPRKE ER

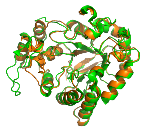

e32b8ed0:epoch_20 - TM score: 0.891, Seq Recovery: 0.33, Visibility: 0.09
DDSIELSNGK KKPKTKQGTG GADEEEAKEA VKDAIKNGIK GIDTGEGNGN NKAVGEAIDE AEKNGDVNED DLDIEDKIGG
DGNRKGKGED AVNKSLEDLN KDKVDVNKNG NSDATNDDGT EDVDEDVTDQ HKEQDKIKKD GKSDSIGTSN ANNDQIDEAE
ATGGDKVDDH DDEINNGNKN DDLIDHCKDK DINVTADKPN GKDDTEDNTD DDGTKGDDTD KESDGNDDNV KKKADEKGKT
ADQINNQYCK DKGCSVEPGE KDKDDIKDNN DDNDFSLTDD EIDDLDKSGK NEKEDKRDEN KGHKNDANKD ED

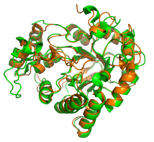

458340e4:epoch_10 - TM score: 0.929, Seq Recovery: 0.39, Visibility: 0.16
TKTIELSNGK KLPILKLGTG GCTEEECKEA VKTAIKNGIK GIDTAKDNGN EKAVGEAIKE CEEEGICKRE DLCIETKIGA
DGLNPGKGKK EVEEMLKELG VDKVDICLAG NCCCKNKDGT EDIDVPVTEI WKELDEIKKE GLCDSIGVSN CNIDQIDECE
KTGGTKIDVV KTEINIYNPQ KELIEHCKEK NINVIADKPL GKKGKKENVK EDGTKEEEED LESEKTDENV KELAKEKGKT
EEQICIKWCI DKGCSVTPGE KKEEDIKEVG KIDDFKLTEE DIKKLEESGK NEIQDKREEN KGHKNDPRKE ER

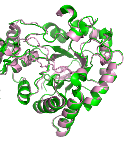

458340e4:epoch_20 - TM score: 0.931, Seq Recovery: 0.38, Visibility: 0.13
TKCIELSNGK KLPIIGLGLG DCTKEEAKEA VKEAIKAGCK CICTASENGN EEAVGEAIKE CIEEGICKRE DLCIITKIGS
ECCRKGKAKE CVDKDLEKLG VDKVDVCLCG NCCCFNEDET ECIDEPITDI WKQMCDIKKE GKADAIGCCN CNNKQIDECE
ATGGEKCDIV KVECNIDNPQ KDLIDYCKKK NIGVICDKPL GREGKECNTK EDGTKGKCKD KESEKTNEEV KKLAKEKGKT
PEQICIKWCI DKGCCVEPEE KDKEKIKDCL KVDDFSLTDE EIEKLEEDGK NEKKDKRDWC KGCKNDPDKD EW

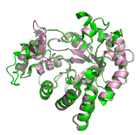

v_48_020 - TM score: 0.923, Seq Recovery: 0.49, Visibility: 1.02
MKTLTLSNGL KMPLLMLGLG GATPEEAVEA VKTAVKAGMR GISTAASYGN EAAVGEAIKE LLEEGVVKRE ELFVETKIPP
HGLRPGRARE TALAMLEELG LDRVDLLLAG NPCAFNEDLT EFVPVPVTEI HRQLDEIKAE GLADAIGVSN ANIEQIEAAL
ALGLTPIDVL RTEINIEYPQ TELIAFCKKH NILVIADRPL GRRGRRRFVL PSGVRLASVP LPSPLTLPEV LALAKELGKT
PEQILIRWVL ERGCAVTPGA REPEAIKENL KVFDFSLTEA DLARLLASGR NELLDKREFT KGHPNDPYKE LR

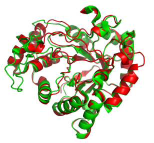

Box 7: **Template and designed sequences for 1QWK**

A.11 1UBQ

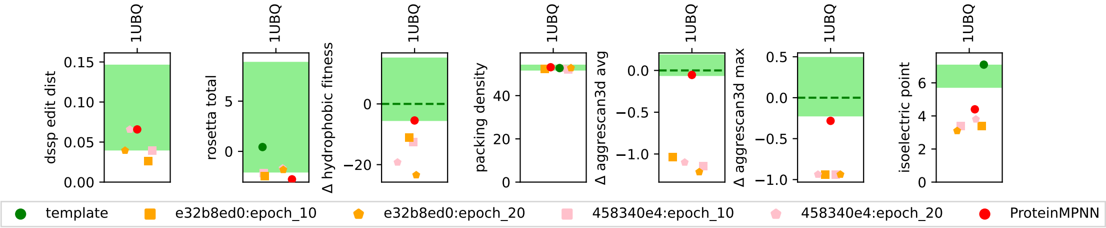

Figure A17: Focus on 1UBQ

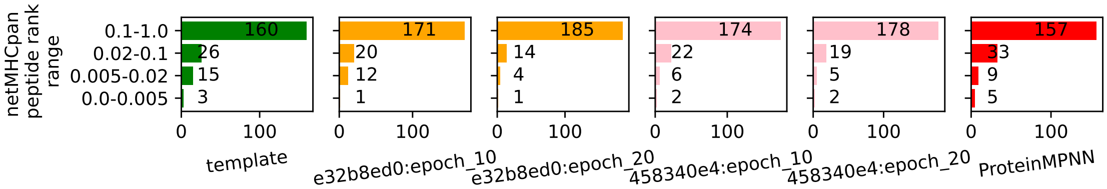

Figure A18: netMHCpan ranks for 1UBQ designs

template
MQIFVKTLTG KTITLEVEPS DTIENVKAKI QDKEGIPPDQ QRLIFAGKQL EDGRTLSDYN IQKESTLHLV LRLRGG

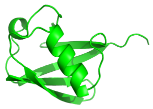

e32b8ed0:epoch_10 - TM score: 0.946, Seq Recovery: 0.49, Visibility: 0.56
MKIKIKDEDG NTIELEVDED DTIAEVKKKI EEKTGIDPEE QKLIYNGEEL EDDKTLKDYN IKEGDTIELE LEDDGE

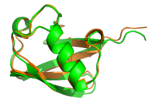

e32b8ed0:epoch_20 - TM score: 0.909, Seq Recovery: 0.42, Visibility: 0.17
MNINVEDEDG NTTELEVDDD DTVEDVKKKI KEKDGTDEDE DKLIYDDEEL EDDDTLDDYD IEEDDTLERE KEKDGG

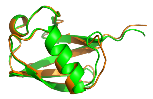

458340e4:epoch_10 - TM score: 0.956, Seq Recovery: 0.45, Visibility: 0.44
MKIKIKDEDG NTIELEVDED DTIEEVKEKI EEETGIDEEE QKLIYKGEEL EDDKTLKDYD IKEGDTIDLE IEDDGE

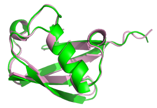

458340e4:epoch_20 - TM score: 0.960, Seq Recovery: 0.47, Visibility: 0.39
MKIKIKGEDG KTIELEVDED DTIEEVKEKI EEKTGIDEEE QKLIYKGEEL EDDKTLKDYD IKEGDTIDLE IEDDGE

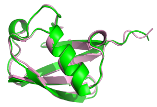

v_48_020 - TM score: 0.960, Seq Recovery: 0.58, Visibility: 0.78
MTIKVKFTDG TTLELEVSPS DTIAELKKKI QEKTGIPPEE QRLIYKGKVL EDDKTLADYN IQEGDTIELE LVPKGG

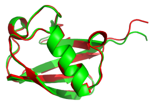

Box 8: **Template and designed sequences for 1UBQ**

A.12 1SQ3

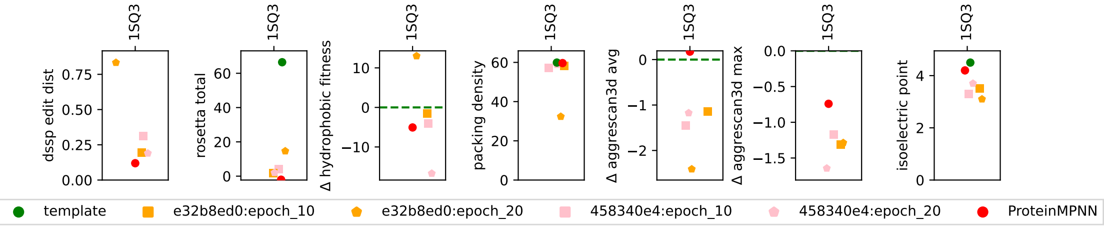

Figure A19: Focus on 1SQ3

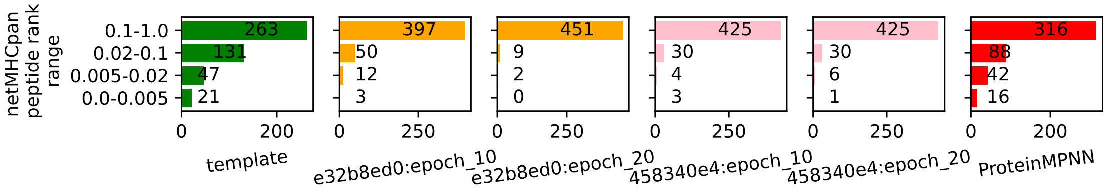

Figure A20: netMHCpan ranks for 1SQ3 designs

template
SISEKMVEAL NRQINAEIYS AYLYLSMASY FDSIGLKGFS NWMRVQWQEE LMHAMKMFDF VSERGGRVKL YAVEEPPSEW
DSPLAAFEHV YEHEVNVTKR IHELVEMAMQ EKDFATYNFL QWYVAEQVEE EASALDIVEK LRLIGEDKRA LLFLDKELSL
RQ

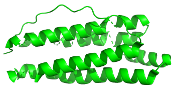

e32b8ed0:epoch_10 - TM score: 0.966, Seq Recovery: 0.43, Visibility: 0.22
MMSEETEEKL NEQLNNERYA GNTEEADASW YRSKGREGFA KEEEVEAAEE LEHADDIADF IEERGGEVKL EQIEQPKQEW
DSPLAAEEDH LEQEEETTEE EEDIVEEAEE EGDDETEEFM QEYVEQQEEE EDEEEEDVDK LKEIGDDKEA EKKLDEEEGE
EE

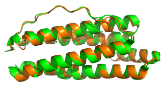

e32b8ed0:epoch_20 - TM score: 0.202, Seq Recovery: 0.28, Visibility: 0.03
DMTDDNNDAE NDQVNNEEQA GKDEDADADW ENDNGNDGEA KEEEVEAEEE QEHADDKEDD IEDRGGDVDE DQIDQQKDDD
DSDLDAEEDD NNDEKDTTNN EDDNVDKAKD DGDDGTADNE QEDTDDQDEE EDDEDDDVDK LKDNGDDKEA KDDNDKEEGK
EE

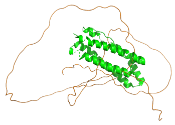

458340e4:epoch_10 - TM score: 0.971, Seq Recovery: 0.36, Visibility: 0.10
MMSEETEEKL NEQVNDERWT GNEEEADASW CKDKGKEGWA KEEEVEAEEE NEHAEDWEDF IEERGGKVKL EQIEQPKEEW
DSELALEEDD LEDEEETTEE EEDIVKEAEE EGDDETEEFN QEDIEDQEEE EDEEEEDVDE LEECGDDKEC QDDEDKEEGE
EE

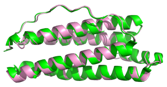

458340e4:epoch_20 - TM score: 0.963, Seq Recovery: 0.37, Visibility: 0.10
KMSEETCKKL NEQINDERWT GNEEEADASW CKSKGKDGWA KEEEVEATEE NGHADDWKDF IEERGGKVEL DKIDKPKQEW
DSELAMEEDA EEGEEETTEE EEDIVKEAEE EGDDETKEFN QEDIEDQEEE EDDEEDDVDK LKECGDDKEC QEELDKEEGK
EK

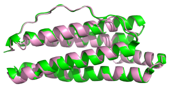

v_48_020 - TM score: 0.988, Seq Recovery: 0.43, Visibility: 0.85
AMSAALVAAL NAQVNAELTA SYTELAAASW YRSHGYDGFA QWEKVAAAEE IAHALALADY IHDRGGTLVL QQIPQPKQTW
ASPLAAEADA LAAEQATTAE VQALVAQAKA AGDTATETFL QAYIAAQVEE VAEEQAIVDK LKAIGDDKAA LLALDKELGK
VK

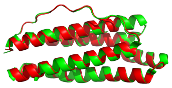

Box 9: **Template and designed sequences for 1SQ3**

A.13 1S5T

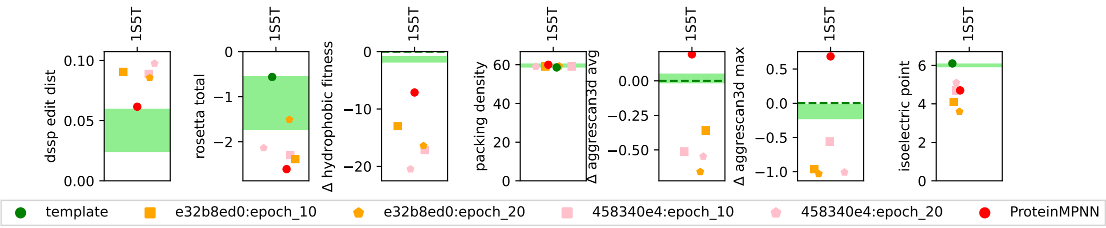

Figure A21: Focus on 1S5T

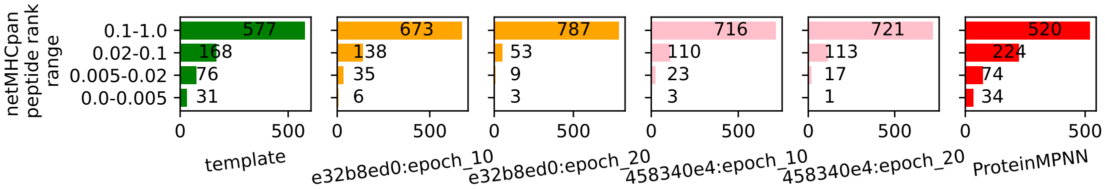

Figure A22: netMHCpan ranks for 1S5T designs

template

MFTGSIVAIV TPMDEKGNVC RASLKKLIDY HVASGTSAIV SVGVTGESAT LNHDEHADVV MMTLDLADGR IPVIAGTGAN
ATAEAISLTQ RFNDSGIVGC LTVTPYYNRP SQEGLYQHFK AIAEHTDLPQ ILYNVPSRTG CDLLPETVGR LAKVKNIIGI
KEATGNLTRV NQIKELVSDD FVLLSGDDAS ALDFMQLGGH GVISVTANVA ARDMAQMCKL AAEGHFAEAR VINQRLMPLH
NKLFVEPNPI PVKWACKELG LVATDTLRLP MTPITDSGRE TVRAALKHAG LL

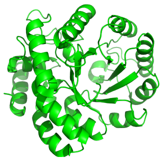

e32b8ed0:epoch_10 - TM score: 0.993, Seq Recovery: 0.52, Visibility: 0.38

MFEGAIAAMI TPRDADGKMD KESLKKMVDR MIEDGIKAIV AVGTTGEADV LDDDERIEVI NTTIELADGK IPVIAGTSAS
DTDEAIALTK KVEGSGVEGI LSGTPGGVKP TQEGLKDHYT AVADNTDLPI ILDNNPARTG TKITPETVGE LSKNENIIGI
KNGSGDLSEV DAIKELVDDD FKILSGTDDT ALDEMKAGGD GSISGTANVA GKDCAEMADL AAKGEYDEAQ KIDDKLKDLN
INKNAEPNPV CTKWAAKELG LIDNDTVRAP YTPATEEAKE KIRAALKKAG KL

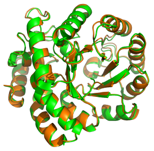

e32b8ed0:epoch_20 - TM score: 0.987, Seq Recovery: 0.43, Visibility: 0.11

MNDGAIAAAT NPKDDDGNDD DDSRKKQVDD DIDNGVDGIV ADGTTGEADT NDDDTAKDNV DKTIDDADGK IPVIAGTGDN
DTDNAIKKAK TVEDSGVDGN DTGNPGGNKD TQDGIKDHYT DVADNTDLPI ITDNKPDRTG TNMEPDTVGE LAKNDNIVGT
KDETGDTDNV DDIKDKVDDD YKILSGNDDT ALDDQAKGGD GNVSGTANVA GKDEAEQADK AADGDNDGAK EIDDKNAKLN
KDKGSEKNPV NTKEGEKTLG KTDSDTVNEP DTPATDEDKE KIDDALKDAG KL

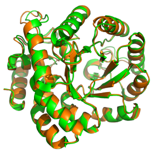

458340e4:epoch_10 - TM score: 0.986, Seq Recovery: 0.46, Visibility: 0.24

MFEGCICAII TPKDKNGNYD EKSMKKMIDK CIEDGIDAIV ACGGTGEKDV LNDEEKKEVV DTTIKEADGK IPVIAGVSDN
DTDKAIEKAK KVEGKGVEGL LCGAPKGIKP TQKGIIDHYT KVADNTDLPI ILDNDPKVNG TDIEPDVVGE LAKNENIIGI
KDSTGKLDNV KAIKEKVGDD FKILSGKDEV ALDAIKEGAD GCISTVCNVA GKDCSEMCKL CQEGKFDEAK KIDDKLKDLK
INFGSQPNPV CTKWACKKLG LIDNDTVNEP YKPATEEDKK KIEEALKKAG KL

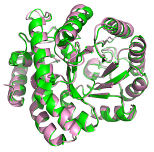

458340e4:epoch_20 - TM score: 0.990, Seq Recovery: 0.47, Visibility: 0.17

MFEGCICACI CPKDEKGKYD EKSCKKIIDK MIEDGIDAIV CCGTTGEADV LNDDERIEVI KTCIKEANGK IPVIAGVGSS
DTEEAIKLTK KVEGTGVEGC LCGVPKGIKP TQEEIIDHYT KVADNTDLPI ILDNDPGKTG TDIKPETIGK LSKNKNIIGI
KNGTGNLDEV EKIKEKVDKD FKILSGKDEV ALDCMKKGGD GCISGVCNVC GKDCAEMCKL CQEGKYDEAK KIDDKLKDCC
KNKNCEKNPV CIKWACKELG LIDNDTVNEP YKPISEEAKK KIKEALKKAG KL

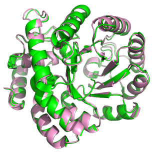

v_48_020 - TM score: 0.993, Seq Recovery: 0.56, Visibility: 1.01

MFTGAIAAIV TPRDAAGNFD RASLARLIDR YIAEGIAALV AVGTTGEAAV LSDEERTAVV LATLELAAGR IPVIAGVGAS
DTEKAIALAK RFEGSGVVGL LLAVPAGVKP SQAGLIAFYT AVADATTLPI ILYNYPALTG TRLEPAAVGE LAKHPNIVGL
KDATGDLANV PAIRALVPAD FRLLSGVDAV ALEYMELGGD GVISAVANVA PAQMAEMAKL AAEGKYEEAK KIADKLAPLA
VALFSQPNPV PIKWALKTLG LIDSDTLRAP YTPATAEDKA TIEAALKAAG LL

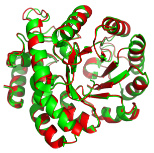

Box 10: **Template and designed sequences for 1S5T**

A.14 1TJE

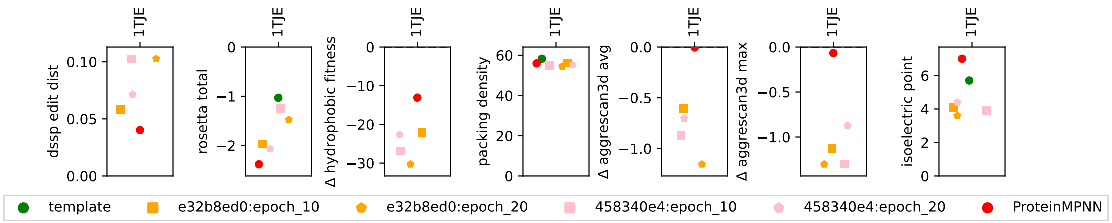

Figure A23: Focus on 1TJE

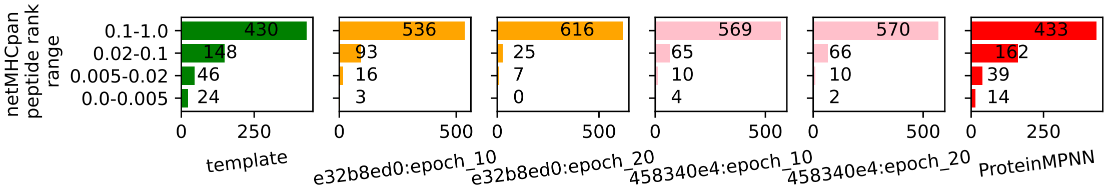

Figure A24: netMHCpan ranks for 1TJE designs

template
MPVITLPDGS QRHYDHAVSP MDVALDIGPG LAKACIAGRV NGELVDACDL IENDAQLSII TAKDEEGLEI IRHSCAHLLG
HAIKQLWPHT KMAIGPVIDN GFYYDVDLDR TLTQEDVEAL EKRMHELAEK NYDVIKKKVS WHEARETFAN RGESYKVSIL
DENIAHDDKP GLYFHEEYVD MCRGPHVPNM RFCHHFKLMK TAGAYWRGDS NNKMLQRIYG TAWA

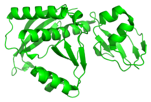

e32b8ed0:epoch_10 - TM score: 0.983, Seq Recovery: 0.48, Visibility: 0.27
MPTITLPDGS TIDKDEPVSI KDIAADIGKD EADKCIAGKA NGELVDADDK IEDDADVEIV TKDDEEGQKI IQHTCGHLVE
AAIKKLYPDT KIGDSPVIDG GFYVDIDTDK DLTDEDIKEI EDECKKIANK DEDIKTETVT KEEAKKIFEA QGDELKVDEL
DNEVDDDEKP TLYKIGDTTT MSDGPVCPNT KFCKNFKLTD KDDRYYKNDK NNKKLQRIKG SAWA

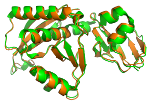

e32b8ed0:epoch_20 - TM score: 0.967, Seq Recovery: 0.42, Visibility: 0.10
APTITLDDGS EIDKDEPVSI DDVAKDIGDD EADKCIGGKA NGENVNADDK IDDDADVEII TDDDEEGKEI IEDTCGDIDN
AAIKEEDPDT KIGDSDTIDG GYETDIDTDK DLTDEDIKKI DDKRKEIADK DEDIENEEVT KDEAKKTFED KGDDDGVDDM
DNNVDDDDKP TENKIGDDTT KGDGPDAPNT KNCKNFKLTD KDDEDKDGDS NNKTKQRIKG SADA

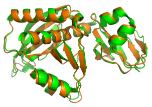

458340e4:epoch_10 - TM score: 0.932, Seq Recovery: 0.46, Visibility: 0.20
MPTITLEDGS KIEKDKPVSV KDIAKDIGKD EADKCIAGKV NGEIVDADEK IEEDADVEII TKDDEEGKKI IQDTCGHLCK
AAIKKIYPDT DIDDCDVIDG GFYCDIDTDK ELTKEDIEKI EKECKEIVNK DEDIKVETVT WEEAKKIFEE KGDEDEVDDL
DDDVDKDEKP TLYKIGDEVT KGDGPVCPNT KFCKNFELTD TDDEYDDGDK DNDKEIRIKG KAWA

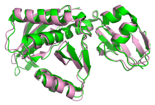

458340e4:epoch_20 - TM score: 0.969, Seq Recovery: 0.43, Visibility: 0.17
GPTIKLEDGS EIECDEPVTI KDIAKSIGEK EEEDCIAGIV NGKKVDADDK IEEDAEVKII TKKDKDGEDI IRDTCADICC
CAIKKIDPDT KIGDCDVIEG GFYCDIKTKK KLTKEDIDKL EKKCKEIANE CKDIKVEKVT WEEAKKIFEE KGNDLKCDKI
DNDIDKDDKP NIYKIGDCIT MGDGPVVPNT CFCKNFELTD ISDKYKDNDK NNEKLQRIKG KAWC

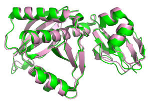

v_48_020 - TM score: 0.980, Seq Recovery: 0.45, Visibility: 0.76
APTITLPDGS TVKADRPVTP AELAASIGAE TAAKLIAGRV NGKLVDASTP IKEDAKVEFI TKDDPEGLKI IQHTCAHLVK
AALKKLYPET IFGEAPVIEG GFYVDVLTKK ELTPEDLKAL EAEIKKIVAK NLPIKVETVT WEEALEIFKA RGDFLQVDRF
LNEVPKDEKP TLYRIGDTVT VSKGPVCPNT KFCQNFKLTD ISYRYYKNDP NNPKLIRIYG SAWA

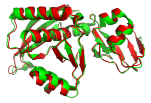

Box 11: **Template and designed sequences for 1TJE**

A.15 1XGD

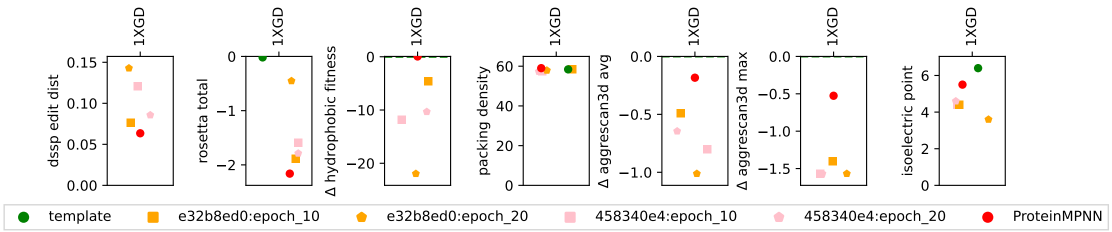

Figure A25: **Focus on 1XGD**

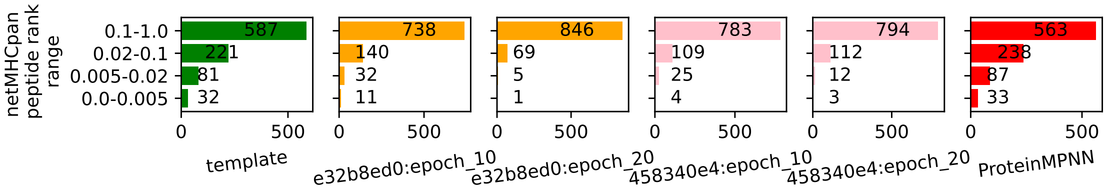

Figure A26: **netMHCpan ranks for 1XGD designs**

template
ASRLLLNNGA KMPILGLGTW KSPPGQVTEA VKVAIDVGYR HIDCAHVYQN ENEVGVAIQE KLREQVVKRE ELFIVSKLWC
TYHEKGLVKG ACQKTLSDLK LDYLDLYLIH WPTGFKPGKE FFPLDESGNV VPSDTNILDT WAAMEELVDE GLVKAIGISN
FNHLQVEMIL NKPGLKYKPA VNQIECHPYL TQEKLIQYCQ SKGIVVTAYS PLGSPDRPWA KPEDPSLLED PRIKAIAAKH
NKTTAQVLIR FPMQRNLVVI PKSVTPEAIA ENFKVFDFEL SSQDMTTLLS YNRNWRVCAL LSCTSHKDYP FHEEF

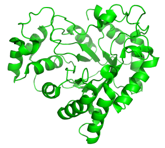

e32b8ed0:epoch_10 - TM score: 0.978, Seq Recovery: 0.44, Visibility: 0.38
MKTIKLNNGN EAPVLQFSTE GVKPEEAEEL VKEAIKEGYK GIKSAKDNGN EAEVGKAINE AIKEGIVKKD ELFIETKIPP
NKHEKDEVEA AVKESLKDLG IDYADLVLRD TPVGLKSGDD LEPKDDNGKP IFSDTTIEET WDAMEELVDK GLAKAIGVSN
FSKEQIDTIL NKEGLKHKPA VNEVERNVEN IQKELTDYCK SKGIVVQSTK PEGDKNDPDK DDDDPGILDD PDIKKIADKH
GKTSSQVIVK YPIEEDEIVA PENTTKEKVK ENKKVFDFKL SDEDIEILNS YDKNRIYDDD SEYKDHPNYP YKTKD

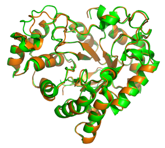

e32b8ed0:epoch_20 - TM score: 0.959, Seq Recovery: 0.38, Visibility: 0.05
NDTLKDNNGN DTPNLEMSTD DVSADEAKDK VKKGIDEGYK GIDSAGDNDN NKEVGDGINE AIDEGKVNED DLNISSEVDN
NDHDKDDVDK AVDDDLKDLG KDKLDNVKKD DPVGKESGDD DDPKDDNGNN KESDTTNNDT WDAMEDTVDD GKANSIGVSN
YNNDQIDDIL NKDGKKNDPA ENEIENNVRN TEKEKNDYDK SKGINVKNDK PDGDDDDDDD DDDDDNIEDD EDIKDIADKK
NKTSKQVLNK YGQEENNKVD PDNTTEEDIE ENNKCNDFEL DDEDKDELDS YDKNERDDDD DDNKDHPDYP NDKDD

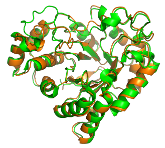

458340e4:epoch_10 - TM score: 0.981, Seq Recovery: 0.46, Visibility: 0.26
KKEIELNNGT KMPTLQFSTE GVEPEKCEEL VKKAIKIGYK GIDTAKDNKN EKEVGKAINE ALKEGIVKED ELCITTKIDN
NDCEKDKVKE AVDKSLKDLG VDKLDLVLRD NPVCLKSGED DEPKDDNGNV IEGDETIEEE WEAMEELVDE GKAKAIGVSN
FNKEQIDKIL NKEGLKNKPA VNEIECNVKN KQKELKKYCK EKGIVCICDK PEGCEDDEDK DEDDPGIKDD PEIKKIADKK
GKTTRQVIIK YPIEEGCIVK PECKTEEKIE ENYKVDDFEL TEEDKEILDS YDKNKICDKD EKYKDHKNYP YKEKD

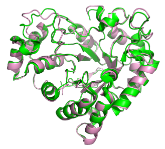

458340e4:epoch_20 - TM score: 0.976, Seq Recovery: 0.42, Visibility: 0.13
GKEIELCNGG KMPVLQFSTE GCPAEKICEL VKKAIKIGYK GIKCAKDNKN EKEVGKAINE CIKEGIVKKD ELCITTKIPP
NCCEKDDVEK CVKDSLKDLG VDDLDLVLRD TPVCLKSGED DEPKDDNGKP ICGDCKIEET WEAMEKCVDN GDCKAIGVCN
FCKEQIDKIL NKEGLKHKPC VNEIECNVKN CQKELIKYCK EKGIVCCCKE PEGCEDDEDK DEDDPGILDD PEIKKIADKK
GKTTRQVCIK WPMEEGCIVC PECKTEEKVE ENNKCFDFEL TEEDKEILDS YDKNKICDKD EKYKDHPDYP YKKKC

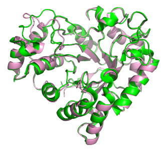

v_48_020 - TM score: 0.968, Seq Recovery: 0.53, Visibility: 1.06
MKEIELNNGL KMPRLLFSTY GVPPEKITEL VKLAIKIGYK GIDSALSYGN EAEVGKAINE ALKEGIVKRE ELFIITKIPP
TRHEPGLVRE AVLEALKELQ VDYLDLVLRH LPVGLKPGEA LFPLDENGKP LFSDVTLLDT WKAMQELVDE GLAKAIGVSR
FSKEQIDTLL NMPGLKYKPA VNRIECNPYN IQKELIEYLK SKGIVVIAYK PLGSPNDPDK SPDDPSLLEH PEILEIAKRH
GKTPRQVLIK YPMERDMIVS PEMTTEEKVR ESYKVFDFKL SEEDLKILDS FDKNRLYDTF PELKDHPLYP YKTKY

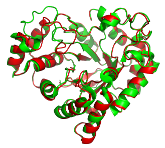

Box 12: **Template and designed sequences for 1XGD**

A.16 1X0M

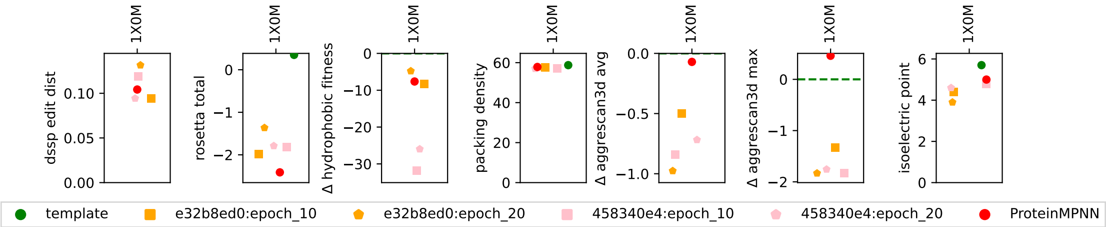

Figure A27: **Focus on 1X0M**

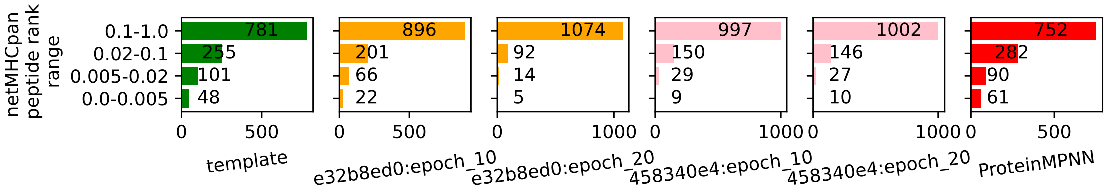

Figure A28: **netMHCpan ranks for 1X0M designs**

```
template
MLGDVERFFS  KKALEMRASE  VRELLKLVET  SDIISLAGGL  PNPKTFPKEI  IRDILVEIME  KYADKALQYG  TTKGFTPLRE
TLMKWLGKRY  GISQDNDIMI  TSGSQQALDL  IGRVFLNPGD  IVVVEAPTYL  AALQAFNFYE  PQYIQIPLDD  EGMKVEILEE
KLKELKSQGK  KVKVVYTVPT  FQNPAGVTMN  EDRRKYLLEL  ASEYDFIVVE  DDPYGELRYS  GNPEKKIKAL  DNEGRVIYLG
TFSKILAPGF  RIGWMVGDPG  IIRKMEIAKQ  STDLCTNVFG  QVVAWRYVDG  GYLEKHIPEI  RKFYKPRRDA  MLEALEEFMP
EGVKWTKPEG  GMFIWVTLPD  GIDSKKMLER  AIKKGVAYVP  GEAFYAHRDV  KNTMRLNFTY  VDEDKIMEGI  KRLAETIKEE
LKA
e32b8ed0:epoch_10  - TM score:  0.988, Seq Recovery:  0.45, Visibility:  0.59
EEGDLSKYLT  EKEKKKKPDP  IEEEKEELAN  TDVIDLGGDE  IDKETIPTED  LKEIEEEIDK  EEKEEATGDG  DTKGDKDLLD
TLKDYYNNTY  GISKDKDIMT  TDGSGDALRL  IGKAYIEPGD  IVVVENPTYP  GALKALEEYE  PKFVEVPVDE  NGMKVDELEE
KLDELKAKGE  KVKICVVNPI  NSNPAGVTMS  EENKEKLLDL  ADKYDFKIVS  EEPYYELRYS  GEKPKLIASL  DKNGNVILIG
GFGKTLSNDL  NVGWIIGKKE  DIEKLIEIKK  KEYGHVDKRS  EVIARKYIEG  GHLDKHIPKQ  NEIYKPKLDK  ALDALDKYFP
EGVTYTKPDG  GLNIWVTLPE  GLDAEELREL  AIEKGVGFTP  GKYFSAKADV  TNTARLNYAD  QKEDDIEEGI  KRLAEAVKEA
QKE
e32b8ed0:epoch_20  - TM score:  0.922, Seq Recovery:  0.35, Visibility:  0.13
DNGNEEKNKT  EKEKKKKEDE  VEKEKNANKN  TDTIDLGGGD  EDDATKDTDN  DDKIDDEIDK  NDKDEADGDG  DTEGDEKLKE
NKKKYDKDTN  GVSDDKDIMT  SDGGGESNKL  IGKAELEEGD  IVATSNPTDK  GSIDAYEEEE  PKYVGIDEDD  NGTDVDELEK
KLEELKDKGE  DVKVDDVNSN  NSNPSGVNDD  DDNKDKLNDL  AKENDFDIVD  DDPDYDNLYD  GDKNTRIAAN  DTDGKTKLIG
SDGKTRDKAL  NVGWIEGDKD  DIEKLVEVKK  EEKGEVDKKS  EVKADKYISG  GDKDKDIPKR  NDANKPKNDA  MNDALDDNAP
DGVTHTKPDG  GDNVWVTEPD  GLDSEENKKD  ADDKGVGYEP  GKNYEADKDT  TNTSRLNYED  QDADDIDKGV  AAYADAVKDA
KDG
458340e4:epoch_10  - TM score:  0.952, Seq Recovery:  0.41, Visibility:  0.26
ENGNEEKEKT  EKEKKEKEDE  IEKRKKENEG  KDTIDLSGGD  IDEETIPKEE  IKKIEEKIKK  EDKEKAEGKG  ETKGNEKLIE
TLKKYKNETD  GISEEKDIIV  TEGSGEANDL  IAKCYIEEGD  IVVISNPCDK  GWIKSFEKYE  PEFIEIDVDD  NGIDVDKLEE
KLEELKKEGK  KVKICCVNPI  NANPTGVTTN  EENKKKLLEL  AKKYDFKIVD  DEPNKDLRYK  GEDETTIASL  DEEGKVILIT
TFDKTLDKKL  NVGAIIGKKE  DIEKLIEEKK  KEKGNVDKKN  EVICEYWLKG  GEKEKWIPKI  NEKLKPKNEA  LQKALDKNCP
EGVTWTKPDG  GTNVWVTLPE  GLNAEELEKK  CEEKGVSFTP  GSYFCAKKDV  KNTCRLNFNE  VKEEDIEKGV  KKLCDCIKEA
KKG
458340e4:epoch_20  - TM score:  0.959, Seq Recovery:  0.40, Visibility:  0.25
EEEDESEEET  EEEKKKEKDK  IEEIKEELEN  TDIIDLGGDE  INKKTIPKED  LEEIEKEIKE  NEEKECTGKG  DTKGDKDLLD
TVKDYLNNKY  GISKDKDIIC  TDGGGDAMDI  IAECYIEKGD  IVCVPNPCNN  DCLKCFKKYE  PKFIEIDIDE  NGINVDKLEE
KLDELKEKGE  KIKICCVCPI  NSNPAGVTMN  EENKEKLIDL  AEKNDFIIVE  DCPRIELQYK  GEKKELIISK  DEKGNVIYIG
TFDKIIDKDL  NVGWIIGKKE  DIKKLIEIKK  KKKGCVNKKN  ECICKKWLEG  GKLDKHIPKI  NKENKPKCEK  LKDALDKNCP
EGVTWTKPDG  GCCVWVTLPE  GLDAEKLREK  CIEKGVGFTP  GKYFCANKDV  TNTCRLNWCD  CEEDDIEKGV  KKFCECIEEA
KKE
v_48_020  - TM score:  0.988, Seq Recovery:  0.47, Visibility:  1.01
SLPDLSLLLS  EEEKKKKPDP  VEERLKAMAN  TSVIDLSGGE  PDPATIPTEI  LKEIEEYIAK  NEKEEATSPG  ETRGYPKLLE
ALKKYLNETY  GISTKFDIMV  TEGTGEAMRL  IGKVLIEPGD  IVVVPAPTYR  GVLRALEPYE  PEFIAIPVDE  NGMIVDELEK
KLEELKAKGK  RVKVVVVNPI  NANPSGVTMS  EERKRKLLEL  AKKYDFIIVE  DLPYYELLYS  GEKPTLIAAL  DEEGRVILLR
TFSKILDPAL  NVGFIYGKPE  LIEKLIEARK  ELYGHVDRYS  EVIAYHYLSG  GYLEKHIPKI  REYLKPKLEA  LLAALKKYFP
EGVTFTTPDG  GLFVWVTLPE  GLDAEKLLEK  AIALGVSFTP  GKYFDPYATV  TNTLRLNFAS  VSPEDIEKGV  EALAKAVKEA
LKK
```

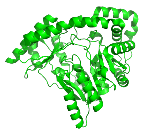

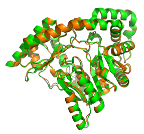

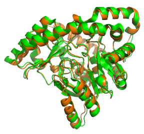

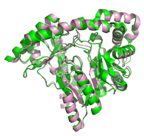

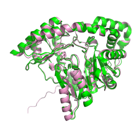

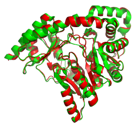

Box 13: **Template and designed sequences for 1X0M**

A.17 2BK8

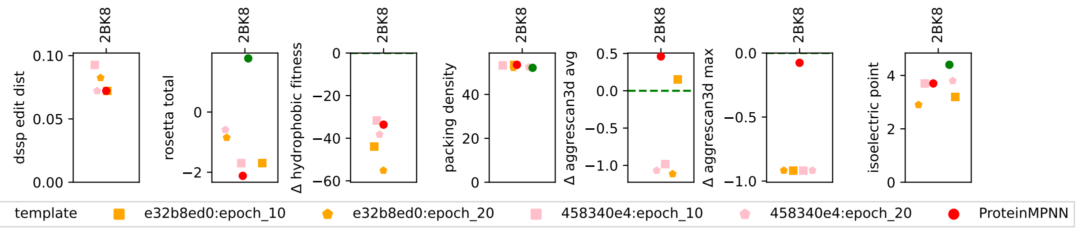

Figure A29: Focus on 2BK8

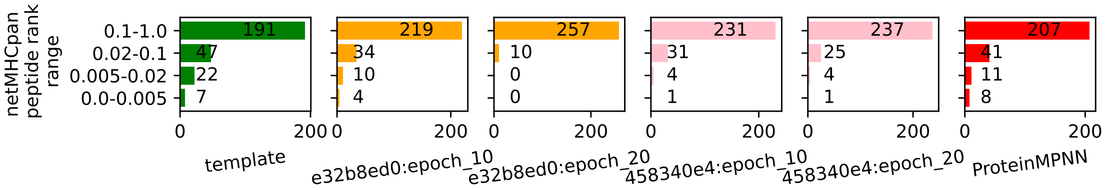

Figure A30: netMHCpan ranks for 2BK8 designs

template
GAMVSGQIMH AVGEEGGHVK YVCKIENYDQ STQVTWYFGV RQLENSEKYE ITYEDGVAIL YVKDITKLDD GTYRCKVVND
YGEDSSYAEL FVKGVRE

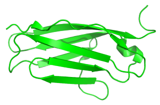

e32b8ed0:epoch_10 - TM score: 0.931, Seq Recovery: 0.43, Visibility: 0.48
SGPTAGSISD QTGTNGGSVT FTCNISGDDD STTVTWSKGS TTLSNSSKYS ISSSSGVSTL TVNNITAADN GTYTCNVTDD
SGSDSASANL TVDGVNA

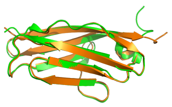

e32b8ed0:epoch_20 - TM score: 0.923, Seq Recovery: 0.39, Visibility: 0.00
GGPTAGDIDD EDCDDGGSVE FTCDIDGDDD TTDVDWKKGD EDLSNDDKND ISDSDGTSKL TVNDCTDDDD GTYTCNVTDD
TGSDSSSGNV DVDGVDD

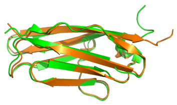

458340e4:epoch_10 - TM score: 0.924, Seq Recovery: 0.54, Visibility: 0.17
GGPKAGKIED KTCEEGGSVT FTCKIEGDDD STKVTWYKGD TELENNDKYD ITYEDGVATL TIKNCTKDDD GTYTCKVEDD
EGSDEAKANI DVEGVDD

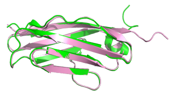

458340e4:epoch_20 - TM score: 0.925, Seq Recovery: 0.55, Visibility: 0.17
GGPKCGKIED KTCEEGGSVT FTCKIEGDDD STKVTWYKGD KELENSDKYD ITYEDGVATL TIKNCTKEDD GTYTCKVEDD
EGSCEAKANI DVEGVDE

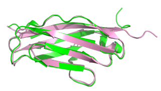

v_48_020 - TM score: 0.949, Seq Recovery: 0.44, Visibility: 0.66
SGPVAGSISD QTGTLGGSVT FTCNISGDNE STTVTWSKGS TTLSNSSKYS ITYSSGVATL TINNITAADN GTYTCNVTDS
SGSDSASAKL TVPGVDP

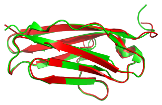

Box 14: **Template and designed sequences for 2BK8**

A.18 2QMT

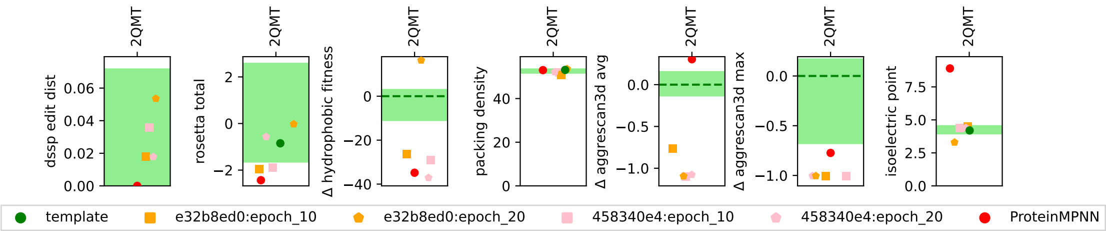

Figure A31: Focus on 2QMT

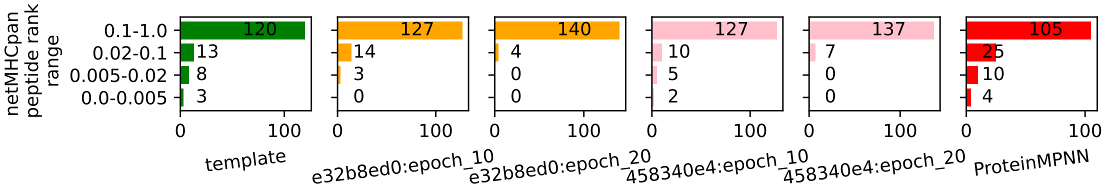

Figure A32: netMHCpan ranks for 2QMT designs

template
MQYKLILNGK TLKGETTTEA VDAATAEKVF KQYANDNGVD GEWTYDDATK TFTVTE

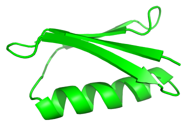

e32b8ed0:epoch_10 - TM score: 0.971, Seq Recovery: 0.52, Visibility: 0.27
ATYTLKINGK NVKGEKSVEA DNAEEAKEKF EKYCKDNGIN GEWTFDEETK TATVTE

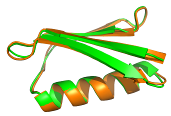

e32b8ed0:epoch_20 - TM score: 0.924, Seq Recovery: 0.45, Visibility: 0.00
ADYTDNTNGK DDKGTTSTDD DNDDDAKDDF DKNNKDNGVD GDETWDDDTK TATSTE

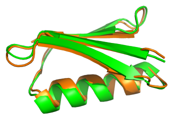

458340e4:epoch_10 - TM score: 0.972, Seq Recovery: 0.50, Visibility: 0.64
AKYKLKIEGK DIKGEKEVEA ENEEEAKEIF EKWCKENGID GEWTYDEETK TFTVKE

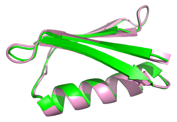

458340e4:epoch_20 - TM score: 0.944, Seq Recovery: 0.52, Visibility: 0.00
GKYKLNINGK EIKGEKECEC ENIEECEKKF KEWCKENGID GEWTCDEETK TFTCTE

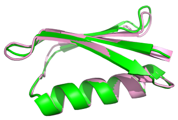

v_48_020 - TM score: 0.974, Seq Recovery: 0.57, Visibility: 1.27
ATYTLNLNGK TVKGTLTVEA ANAAEAQKQF KAYCKSHGIN GTWTFDPSTK TFTVTE

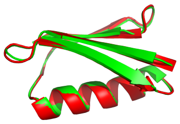

Box 15: **Template and designed sequences for 2QMT**

A.19 3O6A

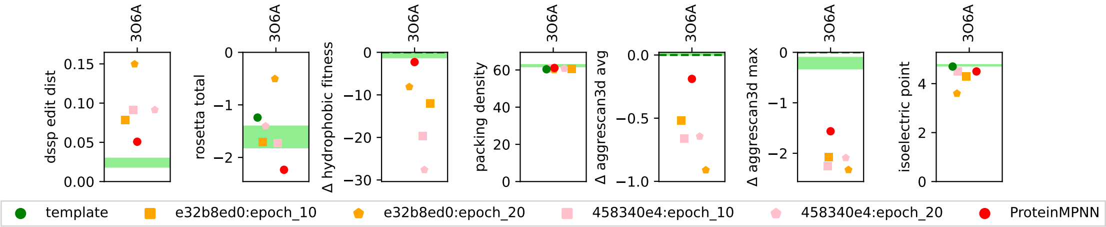

Figure A33: Focus on 3O6A

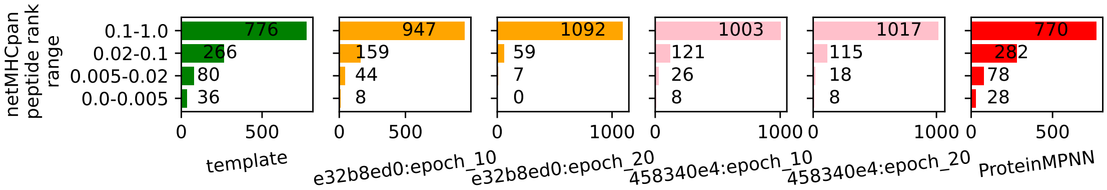

Figure A34: netMHCpan ranks for 3O6A designs

template
AWDYDNNVIR GVNLGGWFVL EPYMTPSLFE PFQNGNDQSG VPVDEYHWTQ TLGKEAALRI LQKHWSTWIT EQDFKQISNL
GLNFVRIPIG YWAFQLLDND PYVQGQVQYL EKALGWARKN NIRVWIDLHG APGSQNGYDN SGLRDSYNFQ NGDNTQVTLN
VLNTIFKKYG GNEYSDVVIG IELLNEPLGP VLNMDKLKQF FLDGYNSLRQ TGSVTPVIIH DAFQVFGYWN NFLTVAEGQW
NVVVDHHHYQ VYSGGELSRN INDHISVACN WGWDAKKESH WNVAGEWSAA LTDCAKWLNG VNRGARYEGA YDNAPYIGSC
QPLLDISQWS DEHKTDTRRY IEAQLDAFEY TGGWVFWSWK TENAPEWSFQ TLTYNGLFPQ PVTDRQFPNQ CGFH

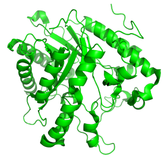

e32b8ed0:epoch_10 - TM score: 0.988, Seq Recovery: 0.48, Visibility: 0.45
NWDWDNDKIK GVNLGGWFVD VPDLRPSRWD KFKDGDDTSN IPKDQEEWDK KKGEEEAKKE RDEWLSTWIT EEDFKEIKDD
GLNHVRIPFG EWEIEKEEND SRVTGGEKYI DKMLEWCKKN NIKANLDLHG VPGSQNGWYD SGESGKLNWQ EGDNRERSLE
VLDKVAKKYC GDKYKDVVIG LETANEPDGS KLDLDKLKEF EKECYDRLKA TGSDTTVVID DAGQETGFWD DFLTEENGET
NVVLSRHYYQ IDSGEKLAKN TDEKIKDICD EGKKITKEKH KSVVGETSAA TDDCCDDYNG VGKGSRTEGK NDGCPYIDDC
EPRKDISNWT DEEKDDIKKY IEAQIEAANA SGGYSFWNWK SENCWENSYK TLKDNGLFPN PIDKKDYPNL CNFE

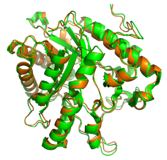

e32b8ed0:epoch_20 - TM score: 0.977, Seq Recovery: 0.34, Visibility: 0.06
NWDWDNDKAN GVNNGGWNIK EADKDKSKYD GEKDGDDDSG ISKDQEEYDK KKGDDKAKEE EDKWRDTKVT KDDYDDIKDD
GLNNVRIDFG DWEIEKDDDD NNVTGGQEKI DEDMENCKDN DIKATLDNHG GSGSNNGGED DGKSGTNGWE DGDNADKDND
VNDKINKEYG GDKNKDVVDG IETENEPDGD KLDQDKVDDY DDKCNKNKKD TGSDTDTEID DNGNDYGADD DKLTEDDGDD
NVVIGRHWGD DNNGDDIAKN DDEKIDDDCK EGEDAKKEKH KNVVTETSSA DDDCCDDENG VGKGSCADGT NDGCDKIDDC
DDKKDEKDDT DDDKENRKKK IEAQIDAANE SGGYDFWDWD SEDCWDNSYK DQKDNGDYPD PIDDKENPDQ CGYD

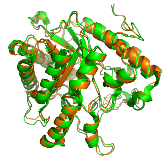

458340e4:epoch_10 - TM score: 0.989, Seq Recovery: 0.46, Visibility: 0.29
NWDWDNDKIK GVNLGGWFVD EPWIRPSRYK KFKKGDDESN IPKDQEEYCK KLGKEKCKEE RDKWLSTWIT EEDFKEIKDD
GLNYVRIPIG EWCIEKEEND SNVTGCEEKL DKCLDWCKKN NIKVNIDLHG CPGSQNGDTD SGKSGVLNWQ EGDNKEKTKE
VLKKLCEKYC GDKYKDVVIG LEVCNEPDNK KLDKDKVKEF EKECYEDLKA TGSDCNVIID DGGEEVGEWD DFLTEEDGCN
NVIINRDYYQ IDSCEKLKKN TDEKIKDICD EGKKRKKEKH KVICGEWSAA TDDCCDWYNG VGKGSCTEGE NNGCEKIDDC
EPKKDIKNWT DKEKDDIKKY IEAQIEACNS FGGYCFWNWK TENCWKNSYK ELKDNGLIPN PIDKKDYPGL CGFE

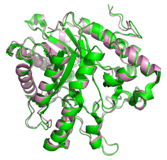

458340e4:epoch_20 - TM score: 0.985, Seq Recovery: 0.44, Visibility: 0.22
NWNWDTDKIK GVNCGGWFVL EPDKRPSLFK PFKKGDDESG IPKDQNEWCK KKGEEECKKE WDKWLSTWIT EEDFKEIKDD
GLNYVRIPIP WCCIEKEEND PWVTGCEEKL DKCLDWCKKN NIKCNIDLHG CPGSQNGDYD SGDSGILNWQ EGDNKEKCKE
CFKKLCEKYC GDKWKDVVIG LECCNEPDGK KLDKDKWKEW CKECYEDLKA TGCDCTCIIH DGGEERGAWD DFLTEEDGCT
NVIIDRDWED IDSCEKIKKN TDEKIKDICD EGKETCKEKH KCICGEWSAA TDDCCDWYNG VGKGSCTEGK NNGCEKIDDC
EPKKDIKNWS DKEKDDIKKY IEAQIEACNK FGGYCFWDWK TENCWKNSYK TLKDNGLIPN PIDKKDYPNL CNFE

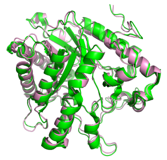

v_48_020 - TM score: 0.990, Seq Recovery: 0.52, Visibility: 0.91
NWDWETDTIR GVNLAGWFVL KPYLRPSLFE PFKVGDDTST IPRDQYEYCK KLGKEKAKEI LDKHWSTWIT AEDFKEIAAD
GLNFVRIPFP YWAIKLLPDD PFVTGEMEYI DKALEWCKEN NIKAILDLHG VPGSQNGWYD SGLSGVLNFQ EGDNLDFALE
VFDIVAKKYC GEKYKDVVIG LELINEPYGA KLDRDKLLAF YERGYNMLKA TGSKVTFIVD DAWQPFGYWD DFLTPADGKE
NVVLSLHFYL IDSNEKLAMT MEEKIAAACA FGREARKLKH KVIFGEFSAA LTDCCPWYFG VGKGSIYEGK YDGGKFIGEC
EPLLDIANWS EQQLEDIRRF IEAQLEAAEA VGGYIFWAWD SENCPLLSYK TLRAAGIYPD PLTARRYPGL CGFP

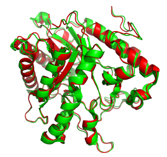

Box 16: **Template and designed sequences for 3O6A**

A.20 3TIP

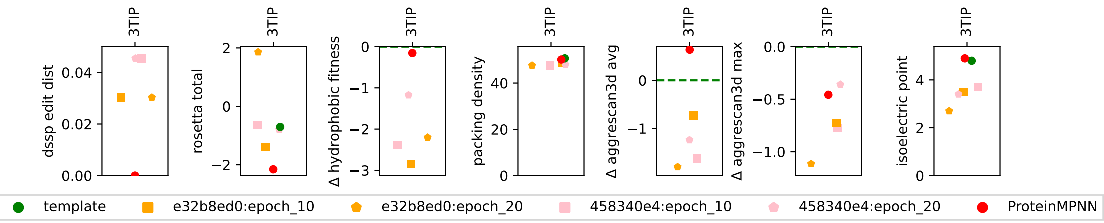

Figure A35: Focus on 3TIP

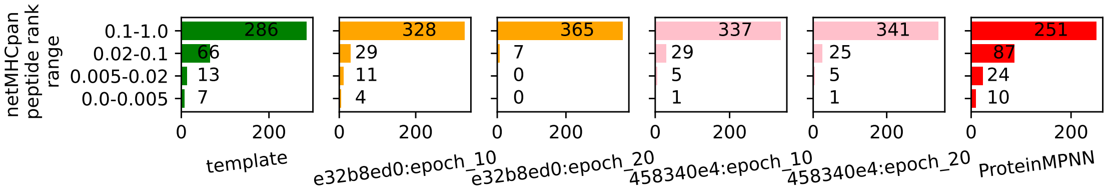

Figure A36: netMHCpan ranks for 3TIP designs

template
GPHMIAPGHR DEFDPKLPTG EKEEVPGKPG IKNPETGDVV RPPVDSVTKY GPVKGDSIVE KEEIPFEKER KFNPDLAPGT
EKVTREGQKG EKTITTPTLK NPLTGEIISK GESKEEITKD PINELTEYGP ET

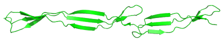

e32b8ed0:epoch_10 - TM score: 0.936, Seq Recovery: 0.51, Visibility: 0.75
GPEKIAPGHE ERFDEDLPVG ESRDVPGEDG IRDPETGEVI KEPTNAVTEY GPEEGEDIEE EETIDDDKTT EKDDDLEAGT
SEVTQEGEDG EKETTTETLK DPLTGEVIEE GEAEEEVVEE PVDEVTKYGD EE

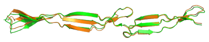

e32b8ed0:epoch_20 - TM score: 0.844, Seq Recovery: 0.31, Visibility: 0.00
GDEKIAPGDT DNDDDDKDTG DTDDNDGTDG VKDPATGETI KDATDANTDN GDDDGDADTD DDDDDDDDDE EDDDDMEDGD
EDVDEDGDDG TDDTTTDTKK DPLTGDNTDD GDATDDTDDD AVDEDTDYGD EE

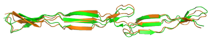

458340e4:epoch_10 - TM score: 0.904, Seq Recovery: 0.46, Visibility: 0.30
GDEKIEPGKK EEFDKDLEVG KEEDIEGENG IKDPETGEVI KEPKDAVTKK GPVKGEDKEE EEDIEDDKEE EEDDEMEAGE
EEVEQEGEDG EKETETETLK NPLTGKVIEE GEAEEEVEEE PVDEVTKEGK EE

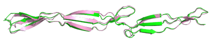

458340e4:epoch_20 - TM score: 0.905, Seq Recovery: 0.45, Visibility: 0.30
GIEKIEPGEE EEFDEDLDVG TEEDIEGENG EKDPETGEVI KEPKNAVKKY GPVEGEDDEE EEDIEDDKEE EEDDEMEAGE
EEVVQEGEDG EKETTTETLK NPLTGEVIDE GEAEEEVVEE PVDEVTKEGK EE

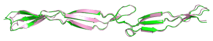

v_48_020 - TM score: 0.880, Seq Recovery: 0.57, Visibility: 1.70
GLEPLPPGHR ERFDPTLPVG ESREIPGRPG LRDPATGRVL RPPVDAVTEY GPVEGEPIVE KREIPAKTTT KYDPSLPPGT
SRVTQEGKPG LKEITTPTLK NPLTGEVIAR GEPVEKVVQP PVDEVIEYGP AS

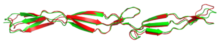

Box 17: **Template and designed sequences for 3TIP**

A.21 3WOY

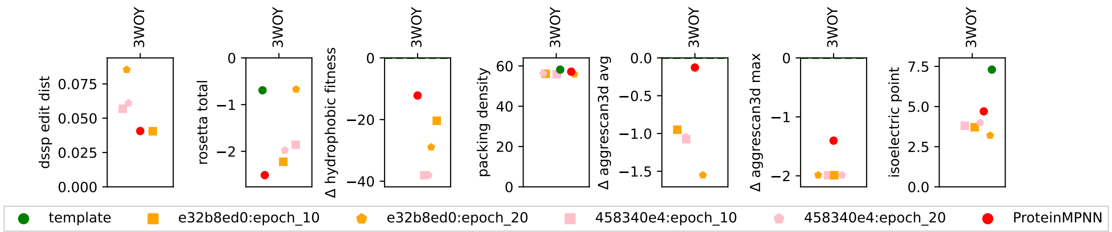

Figure A37: **Focus on 3WOY**

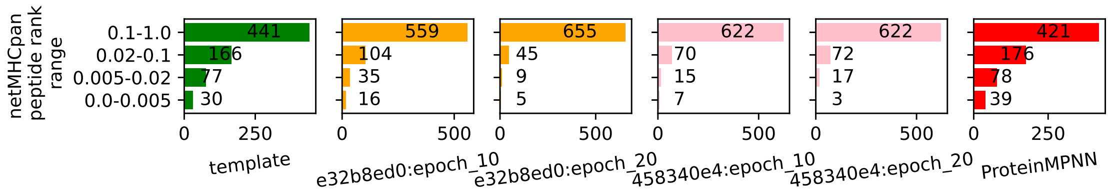

Figure A38: **netMHCpan ranks for 3WOY designs**

template
GAVDEDDFIK AFTDVPSIQI YSSREFLEETL NKIREILSDD KHDWDQRANA LKKIRSLLVA GAAQYDCFFQ HLRLLDGALK
LSAKDLRSQV VREACITVAH LSTVLGNKFD HGAEAIVPTL FNLVPNSAKV MATSGCAAIR FIIRHTHVPR LIPLITSNCT
SKSVPVRRRS FEFLDLLLQE WQTHSLERHA AVLVETIKKG IHDADAEARV EARKTYMGLR NHFPGEAETL YNSLEPSYQK
SLQTYL

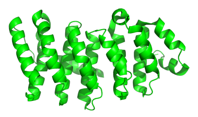

e32b8ed0:epoch_10 - TM score: 0.950, Seq Recovery: 0.36, Visibility: 0.48
MEEEEEEMDE ECDDVEETEI ESEEDLEEEL DEIGEILSDP NTPKEERIEA LREIRSLLNA GAEEYDEFYE ELEELNDELN
NALNDEDPEV VKEAAVTVGC IAKKLGDKAK DTAEAVAPTL LKNVGDEDEE VAEAGITALK EIVKNVKNPN LVPLITDACS
DEDTKVRTEA YRLLAEALDT WDKEDLKDHE DDIADTIKAG VTDEDAAAKE EAKKAFDGLE KHFPEKAKEM RDGLDEDTQK
ELAPAS

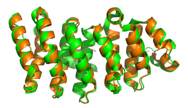

e32b8ed0:epoch_20 - TM score: 0.931, Seq Recovery: 0.26, Visibility: 0.13
DDDDEEEQDK DYDDVDDTEI DSDDDNDDDN DDINDTLDDD NNDKDEQKDA LDDINSNINA GAGDNDSWDD NNDKNNDGLN
KALNDDDSDV VKSGCTTVGA IAKEEGDNAD KTADDVNPTL QDNVGDDDED DAEAGETAIE EINKNSKNKN NIDDITDNTK
DDDEDVRTEA NDDLADALDT WDKEDLEDDK DKINEAVDEG LEDDDEDAKD EANEAFDGVK KHFPDEADET EAKKDEDTKE
DTKDEE

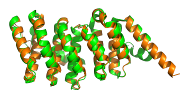

458340e4:epoch_10 - TM score: 0.955, Seq Recovery: 0.30, Visibility: 0.21
EEEEEEEMDE ECDDVEEVEI ESEEDLEEEL DEIKEICSDK NNDEEERIEA LKKICSLINA GAEEYDEFYE ELEELKECLC
NCCEDEDPEV VKECCVCCGC ICKKLKDKAK ELADAIFPVC LKNCGDEDEE KSESGICCCK EIIKNCKNPN LVDKICDACD
DEDTKVRIEA WDLLSEALDT WEKEDLEGNK EKICDCVKKG CEDEDEECKK KAKEAFKGVK KHFPEEAEEM KKGLDEETQE
DLEDEE

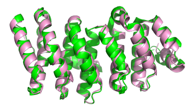

458340e4:epoch_20 - TM score: 0.976, Seq Recovery: 0.31, Visibility: 0.19
KEEEKEECEK EYEDVEEVEI ESEEDLKEEL DEIGDILDDP ETDKEERIEA CKDICSLINA GAGEYDVWKE CLDKLCDCLC
NCCCDEDPEC VKACCVCVGC ICKECGDDFK NCAEKCIPVL IKNVGDENEE KSEAGDVAIE EIIKNCKNPN LIPKICEACE
DENEKVRTKA WDWLATALDT WKKEDLEGNK DKICECIKKG LKDENEECRE EAIKAFKGVK KHFPEEADEM KEELEEEIQE
ECKEEE

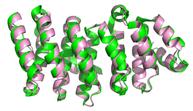

v_48_020 - TM score: 0.974, Seq Recovery: 0.38, Visibility: 1.09
REEVLKEMDK AYTDVPKVTI NSEEDLVKEL DEIGEILADP STPPEERIAA LNRIRSLLNA GAEKYPAFYE RLEKLIPQLQ
LALSSPDPEL RKAAAITVAA IAKTLGERAK KLALAVFPTL LKGLPDPDPT VAEAALAALE EIIKHVHVPE LIPLILDACQ
SPDTRVRIEA YRLLALALET WPKELLLAHE TAIVETIKAG LQDPDPAARE EAIKAFEGLK KHFPEAAAAM LASLPPEIQA
ALAPPS

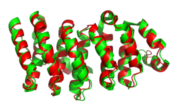

Box 18: **Template and designed sequences for 3WOY**

A.22 4RQG

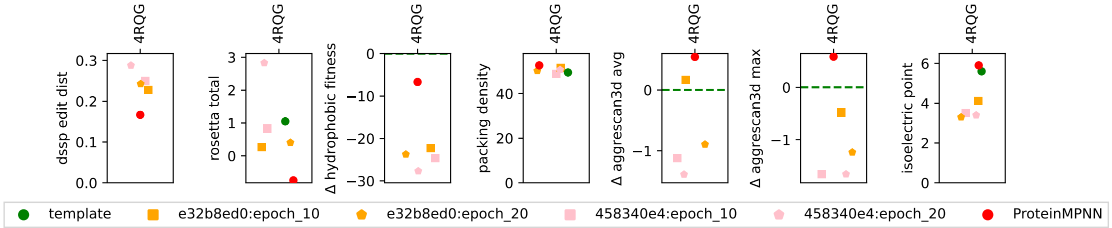

Figure A39: Focus on 4RQG

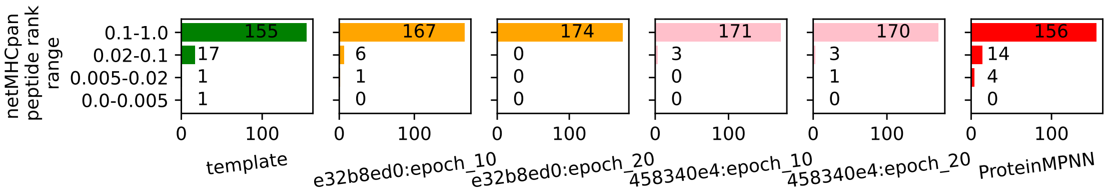

Figure A40: netMHCpan ranks for 4RQG designs

template
ECDCSSPENP CCDAATCKLR PGAQCGEGLC CEQCKFSRAG KICRIPRGDM PDDRCTGQSA DCPRYH

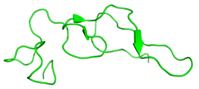

e32b8ed0:epoch_10 - TM score: 0.903, Seq Recovery: 0.49, Visibility: 0.50
ERDCSDSSNP CCDPETGQLK SGCDCCEGPC CSNCKYCAAG TVCRTPTGSA PNDTCTGTCC CCPKVS

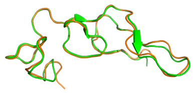

e32b8ed0:epoch_20 - TM score: 0.892, Seq Recovery: 0.46, Visibility: 0.00
DKDCSDDNNP CCDKDTGQLK DGNDCCEGDC CENCQNCDAG TVCDTPTGND KDDTCTGDCC CCPKEN

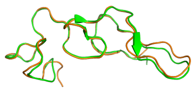

458340e4:epoch_10 - TM score: 0.873, Seq Recovery: 0.55, Visibility: 0.00
ECDCSDDDNP CCDKETCKLK EGCDCCEGEC CENCKYCEKG TVCDEPEGDE PDDTCTGECC CCPKES

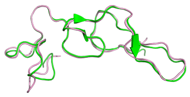

458340e4:epoch_20 - TM score: 0.877, Seq Recovery: 0.50, Visibility: 0.50
ECDCEDDDNE CCDKETCKLK EGCECCEGDC CEDCKYKEEG TICEEPEGDE EEDKCTGDCC CCPEEE

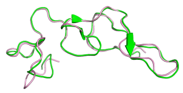

v_48_020 - TM score: 0.913, Seq Recovery: 0.58, Visibility: 2.00
SCDCPDPSNP CCDPATCKLK PGYQCCSGPC CENCKYKPAG TLCRTPTGNE PPDYCTGTCC CCPRVY

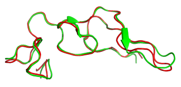

Box 19: **Template and designed sequences for 4RQG**

A.23 4KW4

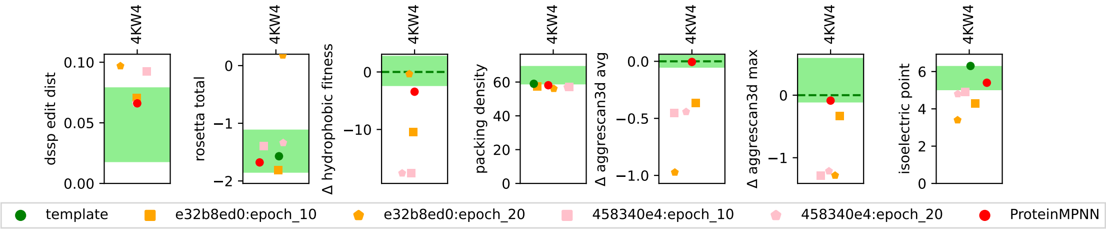

Figure A41: Focus on 4KW4

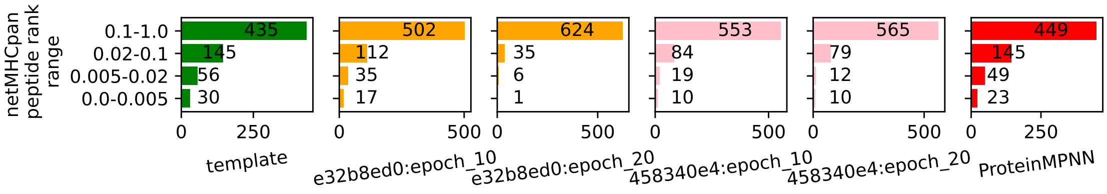

Figure A42: netMHCpan ranks for 4KW4 designs

template
SKGEELFTGV VPILVELDGD VNGHKFSVSG EGEGDATYGK LTLKFICTTG KLPVPWPTLV TTLXXXVQCF ARYPDHMKQH
DFFKSAMPEG YVQERTIFFK DDGNYKTRAE VKFEGDTLVN RIELKGIDFK EDGNILGHKL EYNYNHHKVY ITADKQKNGI
KVNFKTRHNI EDGSVQLADH YQQNTPIGDG PVLLPDNHYL HTHSKLSKDP NEKRDHMVLL EFVTAAGITL

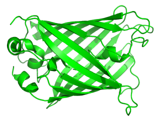

e32b8ed0:epoch_10 - TM score: 0.992, Seq Recovery: 0.68, Visibility: 0.72
SEGAELFKGV VPVVVELDGD VNGKKFSVKG EGEGDATEGR IDLKFVCTTG ELPVPWPTLV TTLXXXIDCF ARYPEHMKDH
DFFKSAMPEG YVRERTNDWE DDGKDDSRGE VKMDGDTLVN RIELKGTDYK EDGNILGHKL KYSAGSSKKN VTADEANNGI
KADFTEELEV EDGSTQKVKN DEKNTPIGDG PVLLPKDHYH ETSAELSKDP NEDRDHMVLR ETIEAGGIEE

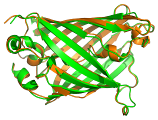

e32b8ed0:epoch_20 - TM score: 0.978, Seq Recovery: 0.50, Visibility: 0.34
SDGDDLFDGK VDIDNDSDGD VNGEKYSNDG EGEGDATNGK TDEDNDNTTG ENPVDWETED DNNXXXIDCN SKYPDHMKDH
DFDKSAMPEG NKDEKDNDDD DDGNDDSRGE VKMDGDTLVN ENEKDGDDYD DDGNILGHKL KDNSGSGKKN IDADEDKNGI
KEDGTDKNDV EDGSTQDSDN DGEKTPIGDG DVKLPDDHYD DTSADNSDDD DEDKDNKKTT ETEEAGGIDE

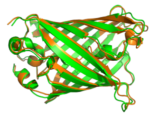

458340e4:epoch_10 - TM score: 0.992, Seq Recovery: 0.64, Visibility: 0.53
SEGEKLFEGK VPIVVEIDGD VNGKKFKVKG EGECDATKGK IDLKCVCTTG KLPVPWPTLV TTAXXXIDCC TKYPEHMKDH
DFFKSCMPEG YVDERTNDWE GDGKDKTRGE VKMDGDTLVN RIELKGTDYK EDGNILGHKL KDSCGSSKKN IKADEKNNGI
KGDFTEELEV EDGSTQKCKN KEKNTPIGDG PVKLPKDHYD ETDCKLSKDK NEKRDHCVIN ETEVAGGIEE

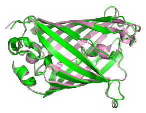

458340e4:epoch_20 - TM score: 0.992, Seq Recovery: 0.61, Visibility: 0.47
CEGGKLFEGK VPIVVECDGD VNGKKFKVKG EGECDATKGK IDLKCVCTTG ECPVPWPTLV TTLXXXIDCC TKYPEHMKDH
DFEKSCMPEG YIDERTNDWE GDGKDKTRCE VKMEGDTLCN RCELKGTDYK EDGNILGHKL KDSCGSSKKN IKADEKNNGI
KKDFTEELEV EDGGVQKCKN KGKNTPIGDG PVKLPKDHYD ETKCKLSKDE NEKRDHCCIE ETEVAGGIEE

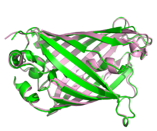

v_48_020 - TM score: 0.986, Seq Recovery: 0.73, Visibility: 0.88
SAGAALFTGV VPVLVELDGD VNGHKFKVVG EGEGDATTGR LVLKFVCTTG KLPVPWPTLV TTLXXXIQCF ARYPPHMAQH
DFFKSAMPEG YVRERTLDVE DDGKFTTRAE VKMEGDTLVN RIELKGTDFK PDGNILGHKL KYDYGSSRVH ITADPARNGI
LAEFTEELPV EDGSTQKVKH KERNTPIGDG PVLLPEPHYH ETAAKLSKDP NEKRDHMVLE ETIVAAGIPE

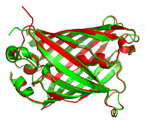

Box 20: **Template and designed sequences for 4KW4**

References

Josh Abramson, Jonas Adler, Jack Dunger, Richard Evans, Tim Green, Alexander Pritzel, Olaf Ronneberger, Lindsay Willmore, Andrew J. Ballard, Joshua Bambrick, Sebastian W. Bodenstein, David A. Evans, Chia-Chun Hung, Michael O'Neill, David Reiman, Kathryn Tunyasuvunakool, Zachary Wu, Akvilė Žemgulytė, Eirini Arvaniti, Charles Beattie, Ottavia Bertolli, Alex Bridgland, Alexey Cherepanov, Miles Congreve, Alexander I. Cowen-Rivers, Andrew Cowie, Michael Figurnov, Fabian B. Fuchs, Hannah Gladman, Rishub Jain, Yousuf A. Khan, Caroline M. R. Low, Kuba Perlin, Anna Potapenko, Pascal Savy, Sukhdeep Singh, Adrian Stecula, Ashok Thillaisundaram, Catherine Tong, Sergei Yakneen, Ellen D. Zhong, Michal Zielinski, Augustin Žídek, Victor Bapst, Pushmeet Kohli, Max Jaderberg, Demis Hassabis, and John M. Jumper. Accurate structure prediction of biomolecular interactions with AlphaFold 3. *Nature*, pages 1–3, May 2024. ISSN 1476-4687. doi: 10.1038/s41586-024-07487-w. URL https://www.nature.com/articles/s41586-024-07487-w.

J. Dauparas, I. Anishchenko, N. Bennett, H. Bai, R. J. Ragotte, L. F. Milles, B. I. M. Wicky, A. Courbet, R. J. de Haas, N. Bethel, P. J. Y. Leung, T. F. Huddy, S. Pellock, D. Tischer, F. Chan, B. Koepnick, H. Nguyen, A. Kang, B. Sankaran, A. K. Bera, N. P. King, and D. Baker. Robust deep learning–based protein sequence design using ProteinMPNN. *Science*, 378(6615):49–56, October 2022. doi: 10.1126/science.add2187. URL https://www.science.org/doi/10.1126/science.add2187.

Noelia Ferruz, Steffen Schmidt, and Birte Höcker. ProtGPT2 is a deep unsupervised language model for protein design. *Nature Communications*, 13(1):4348, July 2022. ISSN 2041-1723. doi: 10.1038/s41467-022-32007-7. URL https://www.nature.com/articles/s41467-022-32007-7.

Sarel J. Fleishman, Andrew Leaver-Fay, Jacob E. Corn, Eva-Maria Strauch, Sagar D. Khare, Nobuyasu Koga, Justin Ashworth, Paul Murphy, Florian Richter, Gordon Lemmon, Jens Meiler, and David Baker. RosettaScripts: A Scripting Language Interface to the Rosetta Macromolecular Modeling Suite. *PLOS ONE*, 6(6):e20161, June 2011. ISSN 1932-6203. doi: 10.1371/journal.pone.0020161. URL https://journals.plos.org/plosone/article?id=10.1371/journal.pone.0020161.

John Jumper, Richard Evans, Alexander Pritzel, Tim Green, Michael Figurnov, Olaf Ronneberger, Kathryn Tunyasuvunakool, Russ Bates, Augustin Žídek, Anna Potapenko, Alex Bridgland, Clemens Meyer, Simon A. A. Kohl, Andrew J. Ballard, Andrew Cowie, Bernardino Romera-Paredes, Stanislav Nikolov, Rishub Jain, Jonas Adler, Trevor Back, Stig Petersen, David Reiman, Ellen Clancy, Michal Zielinski, Martin Steinegger, Michalina Pacholska, Tamas Berghammer, Sebastian Bodenstein, David Silver, Oriol Vinyals, Andrew W. Senior, Koray Kavukcuoglu, Pushmeet Kohli, and Demis Hassabis. Highly accurate protein structure prediction with AlphaFold. *Nature*, 596(7873):583–589, August 2021. ISSN 1476-4687. doi: 10.1038/s41586-021-03819-2. URL https://www.nature.com/articles/s41586-021-03819-2.

Andrew Leaver-Fay, Michael Tyka, Steven M. Lewis, Oliver F. Lange, James Thompson, Ron Jacak, Kristian W. Kaufman, P. Douglas Renfrew, Colin A. Smith, Will Sheffler, Ian W. Davis, Seth Cooper, Adrien Treuille, Daniel J. Mandell, Florian Richter, Yih-En Andrew Ban, Sarel J. Fleishman, Jacob E. Corn, David E. Kim, Sergey Lyskov, Monica Berrondo, Stuart Mentzer, Zoran Popović, James J. Havranek, John Karanicolas, Rhiju Das, Jens Meiler, Tanja Kortemme, Jeffrey J. Gray, Brian Kuhlman, David Baker, and Philip Bradley. Chapter nineteen - Rosetta3: An Object-Oriented Software Suite for the Simulation and Design of Macromolecules. In Michael L. Johnson and Ludwig Brand, editors, *Methods in Enzymology*, volume 487 of *Computer Methods, Part C*, pages 545–574. Academic Press, January 2011. doi: 10.1016/B978-0-12-381270-4.00019-6. URL https://www.sciencedirect.com/science/article/pii/B9780123812704000196.

Suyue Lyu, Shahin Sowlati-Hashjin, and Michael Garton. Variational autoencoder for design of synthetic viral vector serotypes. *Nature Machine Intelligence*, 6(2):147–160, February 2024. ISSN 2522-5839. doi: 10.1038/s42256-023-00787-2. URL https://www.nature.com/articles/s42256-023-00787-2.

Ali Madani, Ben Krause, Eric R. Greene, Subu Subramanian, Benjamin P. Mohr, James M. Holton, Jose Luis Olmos, Caiming Xiong, Zachary Z. Sun, Richard Socher, James S. Fraser, and Nikhil Naik. Large language models generate functional protein sequences across diverse families. *Nature Biotechnology*, pages 1–8, January 2023. ISSN 1546-1696. doi: 10.1038/s41587-022-01618-2. URL https://www.nature.com/articles/s41587-022-01618-2.

Donatas Repecka, Vykintas Jauniskis, Laurynas Karpus, Elzbieta Rembeza, Irmantas Rokaitis, Jan Zrimec, Simona Poviloniene, Audrius Laurynenas, Sandra Viknander, Wissam Abuajwa, Otto Savolainen, Rolandas Meskys, Martin K. M. Engqvist, and Aleksej Zelezniak. Expanding functional protein sequence spaces using generative adversarial networks. *Nature Machine Intelligence*, 3(4):324–333, April 2021. ISSN 2522-5839. doi: 10.1038/s42256-021-00310-5. URL https://www.nature.com/articles/s42256-021-00310-5.

Michael J Stam and Christopher W Wood. DE-STRESS: A user-friendly web application for the evaluation of protein designs. *Protein Engineering, Design and Selection*, 34:gzab029, February 2021. ISSN 1741-0126. doi: 10.1093/protein/gzab029. URL https://doi.org/10.1093/protein/gzab029.
